# An essential role for decorin in bladder cancer invasiveness

**DOI:** 10.1002/emmm.201302655

**Published:** 2013-10-20

**Authors:** Mohamed El Behi, Sophie Krumeich, Catalina Lodillinsky, Aurélie Kamoun, Lorenzo Tibaldi, Gaël Sugano, Aurélien De Reynies, Elodie Chapeaublanc, Agnès Laplanche, Thierry Lebret, Yves Allory, François Radvanyi, Olivier Lantz, Ana María Eiján, Isabelle Bernard-Pierrot, Clotilde Théry

**Affiliations:** 1 Institut Curie Research CenterParis, France; 2 INSERM U932Paris, France; 3 CONICET researcher at Institute of Oncology Angel H. Roffo, University of Buenos AiresBuenos Aires, Argentina; 4 CNRS UMR144Paris, France; 5 Ligue Nationale Contre le Cancer, Cartes d'Identité des Tumeurs programParis, France; 6 Département de Biostatistique et d'Epidémiologie, Institut de Cancérologie Gustave RoussyVillejuif, France; 7 CNRS FRE 2939, Genomes and Cancers, Institut Gustave RoussyVillejuif, France; 8 Service d'Urologie, Hôpital FochSuresnes, France; 9 Université de Versailles – Saint-Quentin-en-Yvelines, Faculté de Médecine Paris – Ile-de-France OuestGuyancourt, France; 10 AP-HP, Groupe Hospitalier Henri Mondor, Département de PathologieCréteil, France; 11 INSERM, Unité 955Créteil, France; 12 Université Paris-Est, Faculté de MédecineCréteil, France; 13 CBT 507, IGR-CurieParis, France

**Keywords:** angiogenesis, bladder carcinoma, decorin, tumour immunity, tumour microenvironment

## Abstract

Muscle-invasive forms of urothelial carcinomas are responsible for most mortality in bladder cancer. Finding new treatments for invasive bladder tumours requires adequate animal models to decipher the mechanisms of progression, in particular the way tumours interact with their microenvironment. Herein, using the murine bladder tumour cell line MB49 and its more aggressive variant MB49-I, we demonstrate that the adaptive immune system efficiently limits progression of MB49, whereas MB49-I has lost tumour antigens and is insensitive to adaptive immune responses. Furthermore, we unravel a parallel mechanism developed by MB49-I to subvert its environment: de novo secretion of the proteoglycan decorin. We show that decorin overexpression in the MB49/MB49-I model is required for efficient progression, by promoting angiogenesis and tumour cell invasiveness. Finally, we show that these results are relevant to muscle-invasive human bladder carcinomas, which overexpress decorin together with angiogenesis- and adhesion/migration-related genes, and that decorin overexpression in the human bladder carcinoma cell line TCCSUP is required for efficient invasiveness *in vitro*. We thus propose decorin as a new therapeutic target for these aggressive tumours.

## INTRODUCTION

Bladder cancer is one of the most common malignant diseases worldwide (Ploeg et al, 2009). About 90% of bladder cancers are urothelial cell carcinomas that arise from bladder epithelial (urothelial) cells. At diagnosis, 20% of patients have muscle-invasive tumours (classified as T2 or higher lesions), the majority being non-muscle invasive (classified as superficial Ta/T1 lesions). Superficial bladder cancers are responsive to immunotherapeutic approaches, such as intravesical instillations of bacillus Calmette-Guerin (BCG) (Lamm et al, 1991). In contrast, muscle-invasive tumours are not responsive to BCG, and 50% of patients relapse after tumour excision and treatment, then presenting with metastatic diseases for which no efficient treatment exist. Hence, the development of alternative immunotherapies would be beneficial for patients bearing muscle-invasive tumours.

Several studies have demonstrated the presence of large amounts of tumour infiltrating lymphocytes (TILs) in both muscle-invasive and superficial bladder tumours. Most of these data have correlated higher numbers of TILs with a good prognosis and survival rate (Ikemoto et al, 1990; Lopez-Beltran et al, 1989; Morita et al, 1990; Tsujihashi et al, 1988) especially in patients with advanced stages of tumour development (Lipponen et al, 1992; Sharma et al, 2007). These TILs are functional and can kill their autologous tumour *in vitro* (Housseau et al, 1997). Although the tumour antigens recognized by these T lymphocytes are not known, next generation sequencing performed on human bladder carcinoma revealed a high rate of non-silent mutations (Gui et al, 2011), which should provide neo-antigens suitable for T-cell recognition. Conversely, we have recently observed that the adaptive immune system may promote bladder carcinoma progression in a mouse model of carcinogen-induced bladder carcinoma, since *Rag2*^*−/−*^ mice devoid of adaptive immune system developed less advanced tumours than immunocompetent hosts (Sugano et al, 2011). Thus, the immune system is not neutral during bladder tumour development and progression, but its exact roles are not clearly understood.

Crosstalk between tumours and non-immune cells creates a particular microenvironment that modulates cancer progression (Lorusso & Ruegg, 2008). The tumour microenvironment contains non-cellular components, especially the extracellular matrix (ECM), composed of a variety of proteins, proteoglycans and polysaccharides (Ozbek et al, 2010). ECM regulates many cellular behaviours and abnormal ECM composition plays a major role in cancer progression (Lu et al, 2012). Decorin, a small leucine-rich proteoglycan of the ECM, is found in the tumour microenvironment and affects the biology of various types of cancer (Iozzo & Sanderson, 2011). While in several studies decorin has been found to have a tumour suppressor role (Csordas et al, 2000; Iozzo et al, 1999; Santra et al, 2000), others correlate decorin with increased tumour invasiveness, metastases and angiogenesis (Benet et al, 2012; Cawthorn et al, 2012; Dil & Banerjee, 2011; Fiedler & Eble, 2009; Zafiropoulos et al, 2008). Decorin is overexpressed in several types of malignancies including ovarian, colon, breast and gastric cancers (Cawthorn et al, 2012; Theocharis et al, 2010). Increased expression of decorin in patients with ovarian cancer is associated with a poor response to treatment and a higher incidence of relapse (Newton et al, 2006). Altogether, these data indicate that depending on the type and context of cancer analysed, decorin can have opposite roles on tumour progression.

Here, we analysed the immune and non-immune tumour microenvironment during progression and invasion of bladder tumours. We took advantage of a classical model of mouse bladder carcinoma cell line, the male MB49 cell line which can be grafted orthotopically in the bladder of syngeneic C57Bl/6J female host (Summerhayes & Franks, 1979), and of its more invasive variant MB49-I recently described by one of our laboratories (Lodillinsky et al, 2009). MB49-I was developed by successive *in vivo* passages of MB49 in syngeneic male hosts, until it displayed more invasive properties than the parental cell line (Lodillinsky et al, 2009). Oversecretion of proteolytic enzymes by MB49-I was observed, and could promote tumour invasiveness, but we asked here whether the immune system and other secreted factors including decorin could also play a role in increased *in vivo* growth of MB49-I.

Our results demonstrate a role for the adaptive immune system in controlling *in vivo* progression of MB49, and escape from this immune surveillance by the invasive MB49-I variant. In addition, we unravel decorin as a new unexpected player in bladder cancer progression, acting both on angiogenesis and on tumour cell invasiveness.

## RESULTS

### The adaptive immune system controls growth of MB49 but not MB49-I

**Figure 1:**
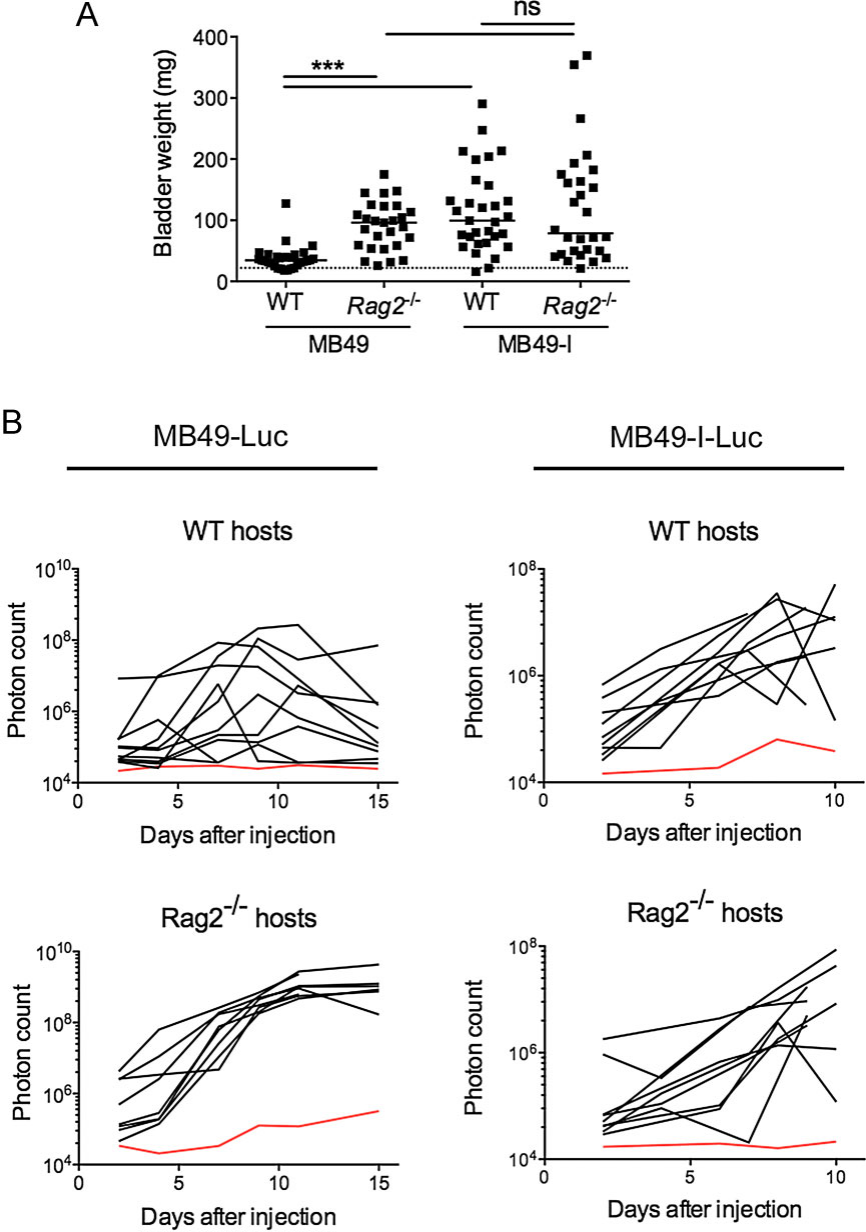
MB49 and MB49-I interact differently with the host immune system

To determine the role of the adaptive immune system in *in vivo* growth of bladder carcinomas, we injected MB49 and MB49-I orthotopically in the bladder of either WT or *Rag2*^*−/−*^ female C57Bl/6J mice, which do not contain any T or B lymphocytes. After 10–12 days (depending on the onset of haematuria in the MB49-I-bearing group), mice were sacrificed and tumour development was quantified by weighing their bladders. As described previously (Lodillinsky et al, 2009), MB49 grew very little in WT hosts, whereas MB49-I developed efficiently. In immuno-deficient hosts, by contrast, the growth difference between MB49 and MB49-I was not significant, because MB49 now gave rise to significantly larger tumours (Fig 1A). These results suggest that MB49 is controlled by the adaptive immune system, whereas MB49-I has developed means to avoid immunosurveillance.

1. Growth of MB49 and MB49-I in WT or *Rag2*^*−/−*^ C57Bl/6J female hosts. Mice were sacrificed 10–12 days after orthotopic injection and weight of individual bladders is shown (pool of 5–7 independent experiments). Dotted line indicates average weight of normal bladders from similarly aged mice. Absence of adaptive immune response promotes MB49 growth, but does not change growth of MB49-I. Horizontal bar = median; ns: non-significant (*p* > 0.9999); ***: *p* < 0.0001 (Mann–Whitney test).
2. Analysis of luciferase activity (expressed as photon count) in WT or *Rag2*^*−/−*^ mice bearing orthotopic MB49-Luc (left panels) or MB49-I-Luc tumours (right panels). Red line indicates background bioluminescence of PBS (non-tumour bearing) mice. Black lines correspond to individual mice pooled from 2 independent experiments. Luciferase activity transiently increases and then decreases only in WT mice bearing MB49-Luc, suggesting adaptive immune system-dependent regression of this tumour.

To achieve longitudinal monitoring of MB49 and MB49-I tumour growing inside bladders, both cell lines were stably transfected with a plasmid encoding firefly luciferase (Luc). In 8 out of 10 WT mice which had received MB49-Luc, the luciferase signal increased and then regressed during the 15 days of follow up, whereas in 8/8 *Rag2*^*−/−*^ mice bearing MB49-Luc, the luciferase signal increased steadily from day 4 onwards without showing the regression pattern (Fig 1B; left panels). In contrast, in both WT and *Rag2*^*−/−*^ host mice, most MB49-I-Luc-bearing mice showed a progressive increase in luciferase activity during time (Fig 1B; right panels). These results confirm that the adaptive immune system controls the development of MB49, whereas it does not affect MB49-I.

### MB49 and MB49-I display different patterns of infiltrating B and T lymphocytes

**Figure 2:**
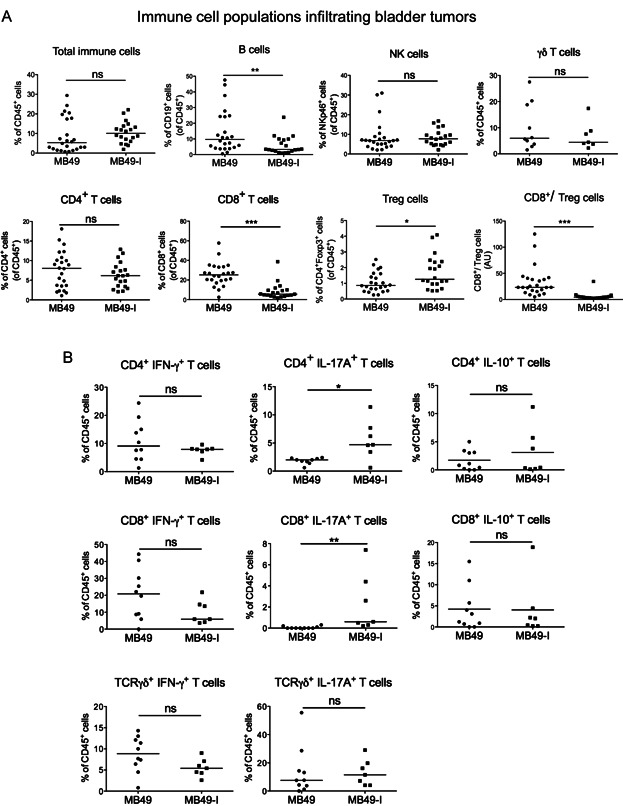
Pattern of lymphoid cells infiltration and cytokine secretion in MB49 and MB49-I tumours

We analysed adaptive immune responses taking place within the growing tumours, first by analysing immune cells infiltrating MB49 and MB49-I in WT hosts. Single cell suspensions were prepared from MB49 and MB49-I tumours growing *in vivo* in bladders, and analysed by multicolour flow cytometry after surface staining for the presence of CD19^+^ B cells, NK cells, CD4^+^ T cells, CD8^+^ T cells, CD4^+^/Foxp3^+^ regulatory T cells (Treg) (Fig 2A), and after intracellular staining for production of cytokines by T cells (Fig 2B). At day 5 post-injection, when both tumours showed progressive growth indicated by increase in luciferase activity (Fig 1B), we did not find any differences in infiltrating immune cells (Supporting Information Fig S1). In contrast, analysis of immune response at days 10–12 post-injection, when MB49 tumours were regressing and MB49-I still growing, showed marked changes (Fig 2). The overall percentage of total immune cells (*i.e*. CD45^+^ cells) infiltrating MB49 or MB49-I tumours was similar, and five times higher than in control (PBS) bladders, which had not received tumours (Fig 2A). Among CD45^+^ cells, CD8^+^ T cells and B cells were present at higher frequency in MB49, whereas regulatory CD4^+^/Foxp3^+^ cells were more abundant in MB49-I, leading to a very high ratio of cytotoxic to regulatory T lymphocytes in MB49 but not in MB49-I tumours (Fig 2A). In contrast, we did not observe any difference in NK cells or in γδ T lymphocytes infiltrating MB49 *versus* MB49-I (Fig 2A).

1. Nature of immune lymphoid cells infiltrating MB49 and MB49-I growing in the bladder of C57Bl/6J female mice. Single cell suspensions were prepared from bladders 10–12 days after injection of the tumour cells and analysed by multicolour flow-cytometry. Immune cells were defined by their expression of the pan-hematopoietic marker CD45 (first panel). Percent of each immune cell type among the total immune cells in individual mice is indicated (pool of 5–7 independent experiments). Horizontal bar = median. ns = non-significant (total cell: *p* = 0.1116; NK cells: *p* = 0.4511; γδ T cells: *p* = 0.5530; CD4^+^ T cells: *p* = 0.3896). *: *p* < 0.05 (Treg cells: *p* = 0.0216); **: *p* < 0.001 (B cells: *p* = 0.0052); ***: *p* < 0.0001 (CD8^+^ T cells; CD8^+^/Treg cells) (Mann–Whitney test).
2. Secretion of IFN-γ, IL-17A and IL-10 by T lymphocytes infiltrating MB49 and MB49-I tumours was analysed by intracellular FACS staining after *in vitro* restimulation. Percent of each cell type in individual mice is indicated (pool of 2 independent experiments). Horizontal bar = median. ns = non-significant (CD4^+^ IFN-γ^+^ T cells: *p* = 0.5839; CD8^+^ IFN-γ^+^ T cells: *p* = 0.1065; TCRγδ^+^ IFN-γ^+^ T cells: *p* = 0.0583; CD4^+^ IL-10^+^ T cells: *p* = 0.6507; CD8^+^ IL-10^+^ T cells: *p* = 0.8697; TCRγδ^+^ IL-17A^+^ T cells: *p* = 0.5831). *: *p* < 0.05 (CD4^+^ IL-17A^+^ T cells: *p* = 0.0126); **: *p* < 0.001 (CD8^+^ IL-17A^+^ T cells: *p* = 0.0003) (Mann–Whitney test).

After *in vitro* restimulation, we observed a tendency for an increased proportion of IFN-γ^+^ cells in CD4^+^, CD8^+^ and γδ T lymphocytes recovered from MB49, whereas CD4^+^ and CD8^+^ T lymphocytes present in MB49-I were significantly more prone to secrete IL-17A (Fig 2B). In contrast, there was no difference in IL-10 producing cells (Fig 2B). Since these differences were observed only 10–12 days after tumour implantation, and not at an earlier time point (d5, Supporting Information Fig S1), they may simply reflect a difference of immune infiltration in a regressing (MB49) *versus* a growing (MB49-I) tumour. Given that MB49 regression does not take place in mice devoid of T and B cells (Fig 1), the pattern of immune infiltrate thus suggest an efficient anti-tumour cytotoxic CD8^+^ T-cell response against MB49, and an impairment of the immune response by regulatory T cells and possibly some IL17A-expressing CD4^+^ (Th17) and CD8^+^ (Tc17) lymphocytes in MB49-I tumours.

### MB49-I has lost ability to activate H-Y-specific CD4^+^ and CD8^+^ T cells

To specifically evaluate the immune response of T lymphocytes against tumour antigens, we used the fact that MB49 expresses minor histocompatibility male antigens from the Y chromosome (H-Y), since it has been established from carcinogen-treated male C57Bl bladder epithelial cells (Summerhayes & Franks, 1979). H-Y antigens are not expressed in female mice (neither adult nor in the thymus during development), and can be targets of the female adaptive immune system (Simpson et al, 1997). H-Y thus represents a prototypical model for neo-antigens resulting from mutations in patient's tumours (*e.g*. point mutations, frameshifts or gene fusions), which create mutated proteins displaying epitopes that can be recognized by the immune system.

**Figure 3:**
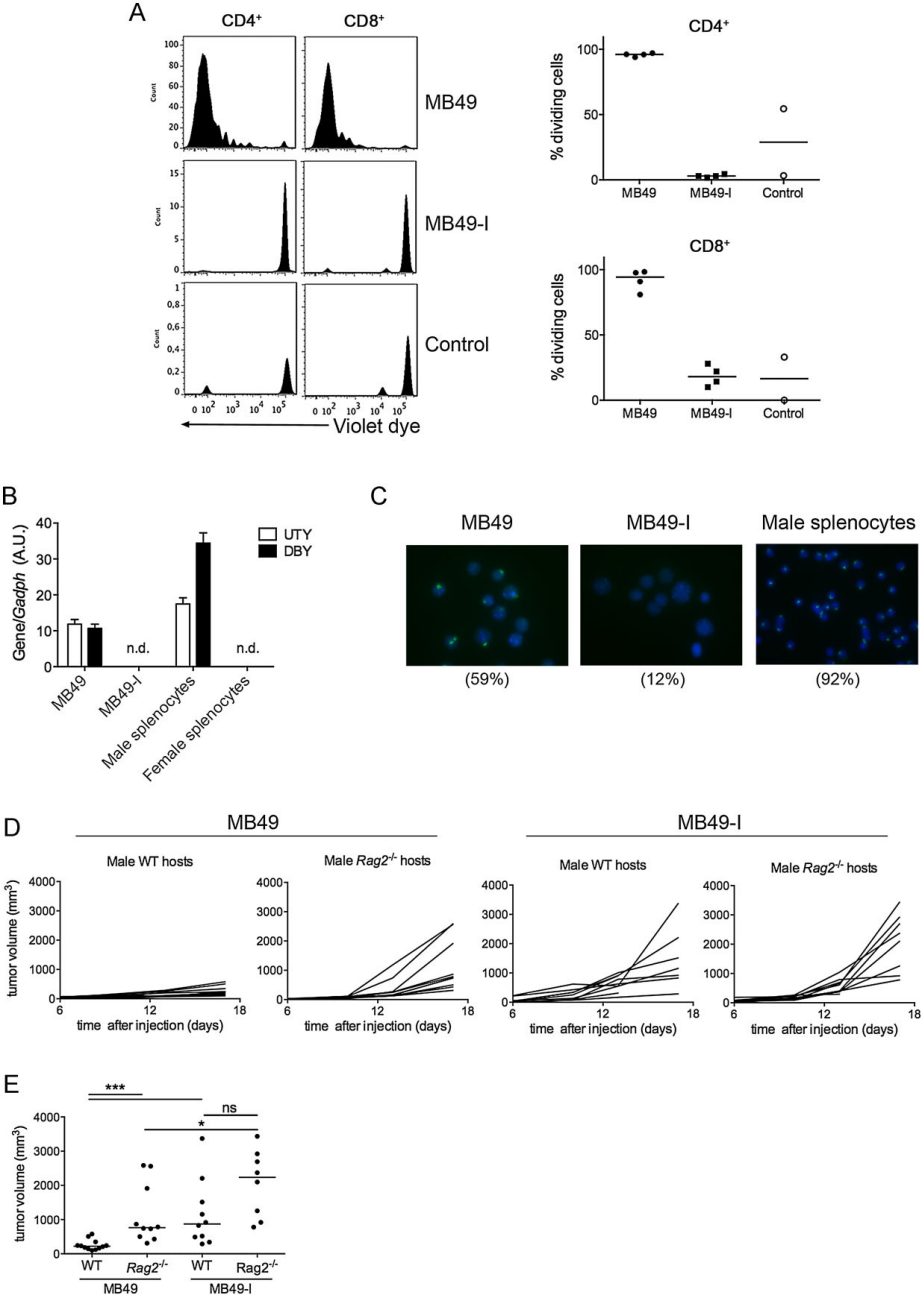
MB49-I is not recognized by H-Y antigen-specific T cells

Presentation of tumour H-Y antigens to CD4^+^ and CD8^+^ T cells can be measured using H-Y-specific T cells from transgenic mice expressing a single TCR recognizing I-A^b^-Dby [Marilyn mice (Lantz et al, 2000)], or D^b^-Uty [MataHari mice (Valujskikh et al, 2002)], respectively. Monoclonal H-Y-specific T cells were transferred to C57Bl/6J mice one day after injection of MB49 or MB49-I in the footpads. Extensive division of both Marilyn (CD4^+^) and MataHari (CD8^+^) T lymphocytes was observed in popliteal lymph nodes of MB49-injected mice, whereas no division was found in mice bearing MB49-I (Fig 3A). These results were not due to induction of a suppressive environment preventing efficient T-cell activation by MB49-I, since Marilyn and MataHari T cells still did not proliferate in mice injected with necrotic MB49-I emulsified with CFA (Supporting Information Fig S2). These results suggested that this variant of MB49 has lost intrinsic expression of the H-Y antigens Uty and Dby recognized by the T lymphocytes. Quantitative RT-PCR analysis of *Uty* and *Dby*, confirmed the lack of expression of both in MB49-I cells, whereas both were expressed in MB49 (Fig 3B). Finally, fluorescence *in situ* hybridation (FISH) using whole Y chromosome probes showed that most MB49-I cells do not display any parts of the Y chromosome, whereas a majority of MB49 are labelled with the probe (Fig 3C). Altogether these data demonstrate that MB49-I has lost expression of antigens present on the Y chromosome, and thus does not activate adaptive immune responses elicited to the male antigen in female hosts.

A. Proliferation of transferred H-Y-specific CD4^+^ and CD8^+^ T lymphocytes was analysed in female CD45.1^+^ C57Bl/6J mice 6 days after footpad injection of MB49 or MB49-I cells, or PBS (control). Five days after T-cell transfer, mice were sacrificed and proliferation was shown by dilution of Violet Dye staining in CD45.2^+^ Vβ6^+^CD4^+^ (Marilyn) or Vβ8^+^CD8^+^ (MataHari) T cells from popliteal lymph nodes (left panels). Quantification of the % of dividing Marilyn and MataHari cells from two independent experiments in shown in right panels.
B. Expression of *Dby* and *Uty* in MB49 and MB49-I cells and in female and male splenocytes by quantitative RT-PCR analysis. Bars represent mean ± SEM of triplicate wells from one experiment (n.d.: not detected).
C. FISH analysis of Y chromosome in MB49, MB49-I and in male splenocytes using a whole chromosome-painting probe. DNA was visualized with DAPI. Numbers in brackets indicate percentage of Y-positive cells in 3 to 4 fields analysed for each cell type.
D,E. Subcutaneous growth of MB49 and MB49-I in WT and *Rag2*^*−/−*^ male hosts. (D) Tumour growth, monitored twice weakly, is represented in individual mice (pool of 2 independent experiments). (E) Quantification of tumour volume at day 17 post-injection is shown. Horizontal bar = median. ns: *p* = 0.6574; *: *p* = 0.0266; ***: *p* < 0.0001 (Mann–Whitney test). MB49 grows more in *Rag2*^*−/−*^ than WT hosts, whereas MB49-I grows similarly in the two strains of mice.

To determine whether loss of H-Y antigens fully explains the higher *in vivo* growth capacity of MB49-I, we compared MB49 and MB49-I development in male mice, which are tolerant to H-Y antigens. Because males have a longer urethra than females, orthotopic implantation in male bladder is not possible. Experiments were thus performed by injecting cells subcutaneously in male WT or *Rag2*^*−/−*^ mice. Fig 3D shows that, as observed in female mice (Fig 1A), growth of MB49 tumour was increased in *Rag2*^*−/−*^ hosts compared to WT hosts, and was only slightly less efficient than MB49-I growth. Thus, adaptive immune response to the H-Y antigens is not the only reason for impairment of MB49 growth *in vivo* in immunocompetent hosts. These results suggest that MB49-I has either lost general ability to activate adaptive immune cells in an antigen non-specific manner, or, more likely, that a variety of tumour antigens expressed by MB49 have been lost during *in vivo* passages leading to establishment of MB49-I.

### MB49 and MB49-I cells secrete differently a few proteins

**Figure 4:**
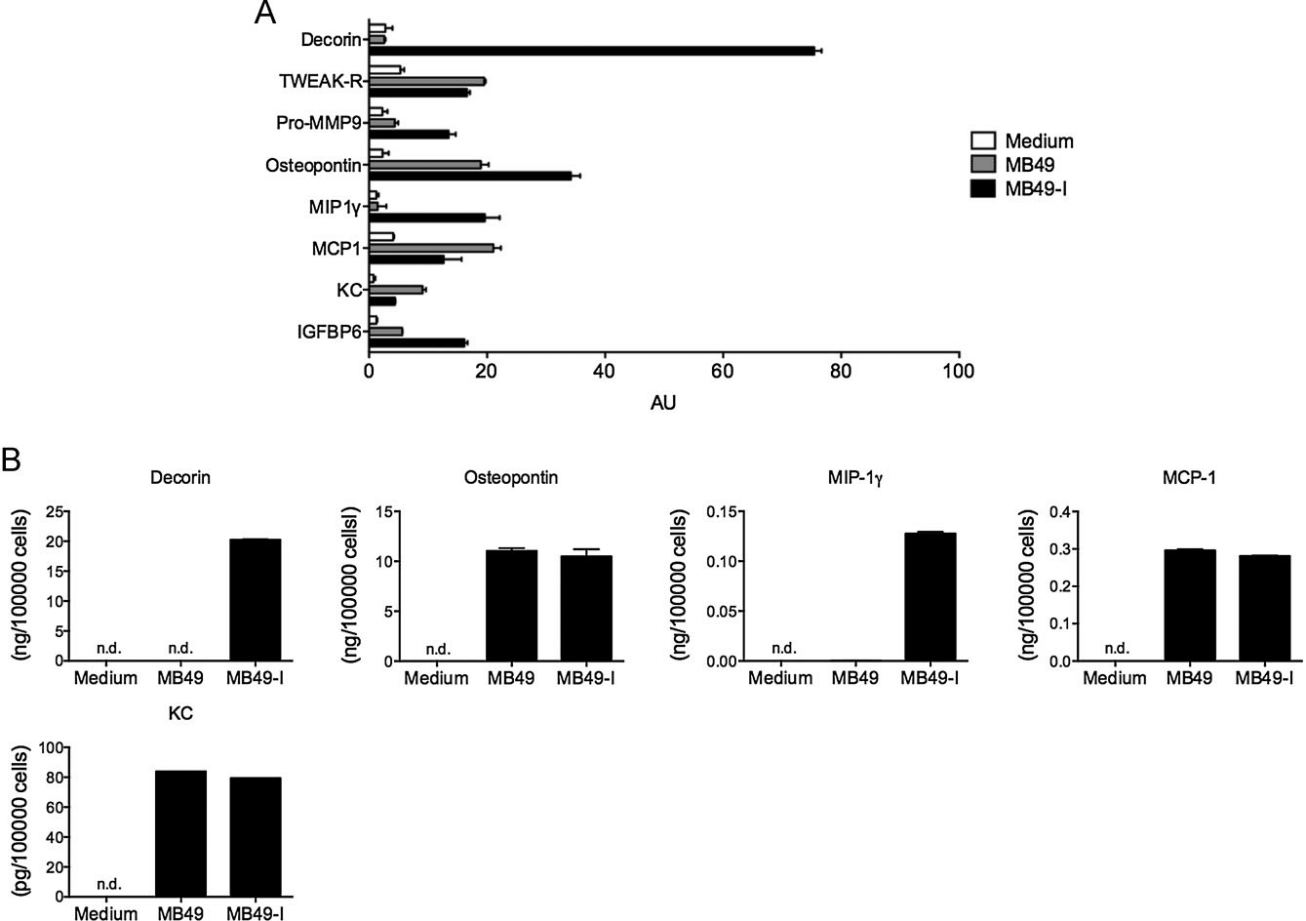
Differential secretions by MB49 and MB49-I cells

Even in the absence of an immune response (in male *Rag2*^*−/−*^ hosts), we observed that MB49-I grew faster than MB49 (Fig 3D), whereas MB49-I divided slower than MB49 *in vitro* (Supporting Information Fig S3). Thus, MB49-I must have developed other mechanisms to subvert its *in vivo* microenvironment, in addition to antigen loss. We therefore analysed the secretion of 144 proteins in MB49 and MB49-I cell culture supernatants using a commercial antibody microarray. Among all proteins analysed (as previously listed in (Bobrie et al, 2012)), 8 were detected in cell supernatants above the level obtained with control medium and all of them except TWEAK-R seemed differently secreted by MB49 and MB49-I cells (Fig 4A). Decorin, matrix metalloproteinase 9 (pro-MMP9), Osteopontin, MIP-1γ and Insulin Growth Factor Binding Protein 6 (Igfbp6) were more abundant in medium conditioned by MB49-I while KC/Cxcl5 and MCP-1/Ccl2 were more secreted by MB49 (Fig 4A). To confirm the semi-quantitative results obtained by antibody array, we quantified secretion of five of these proteins (decorin, osteopontin, KC, MIP-1γ and MCP-1) in MB49 and MB49-I cell supernatants by ELISA. Decorin and MIP-1γ were not detectable in the conditioned medium from MB49, whereas both were readily quantified in the conditioned medium from MB49-I, thus confirming the differential expression evidenced by the antibody array experiment (Fig 4B). By contrast, accurate quantification of osteopontin, KC and MCP-1 did not show any difference in secretion by MB49 and MB49-I, and thus did not confirm the less than twofold difference observed in the antibody array experiment (Fig 4B).

1. Antibody Microarray analysis of protein secretions by MB49 or MB49-I cells *in vitro*, as compared to non-conditioned medium (medium). Proteins that were detected above the background level of medium are shown. Bars represent mean ± SEM of duplicate samples from one experiment.
2. Decorin, Osteopontin, MIP1-γ, MCP-1 and KC were quantified by ELISA in 48 h-conditioned medium of MB49 and MB49-I. (n.d.: not detected). One representative experiment out of 2 is shown.

### Decorin overexpression is necessary and sufficient to explain the enhanced invasiveness of MB49-I

**Figure 5:**
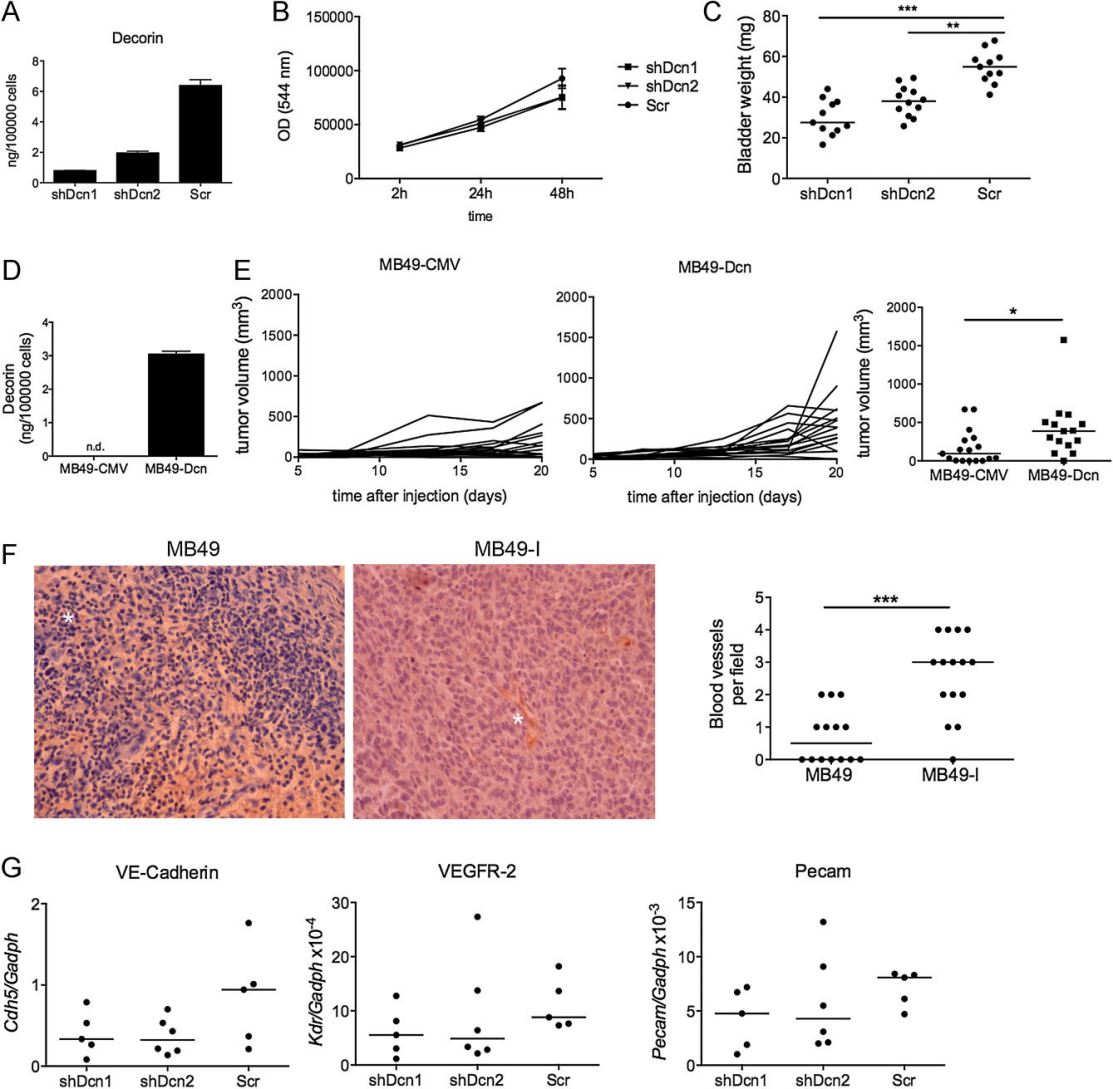
Decorin is necessary and sufficient for increased invasiveness of MB49-I cells

Since MB49-I secreted more than ten times higher amounts of decorin (around 20 ng/10^5^ cells) than MIP-1γ (0.12 ng/10^5^ cells) (Fig 4B), we focused on this protein and tested whether decorin could play a role in MB49-I *in vivo* growth properties. We stably knocked-down decorin expression in MB49-I cells using lentivirus-delivered shRNA and injected them into the bladder of female C57Bl/6J mice. Lentiviruses encoding shDcn1 or shDcn2 strongly downregulated decorin secretion (Fig 5A), without affecting *in vitro* growth of cells (Fig 5B). When injected *in vivo*, MB49-I cells transduced with either shDcn1 or shDcn2 showed decreased growth compared to MB49-I cells transduced with control shRNA (Scr) (Fig 5C). We noticed in two independent experiments that growth of MB49-I-shDcn1 and MB49-I-shDcn2 correlated with their level of secretion of decorin, which indicates that decorin promotes MB49-I tumour growth in a dose-dependent manner (Fig 5A and C). Conversely, we expressed decorin in MB49, and obtained a new MB49 cell line (MB49-Dcn). *In vivo* growth of MB49-Dcn after subcutaneous injection in C57Bl/6J male hosts was significantly increased when compared to the control cell line transfected with an empty pCMV plasmid (Fig 5E). Altogether these findings demonstrate that decorin is at least partly responsible for the increased *in vivo* progression of MB49-I cells.

1. Secretion levels of decorin quantified by ELISA in the 24 h-conditioned medium of MB49-I cells infected with lentiviruses expressing 2 different shRNA to decorin (shDcn1 and shDcn2) or a control shRNA (Scr). Representative results from 2 independent experiments are shown.
2. *In vitro* growth of MB49-I cells transduced with shDcn1, shDcn2 or Scr quantified during 48 h using Cell Titer blue viability assay. (pool of 2 independent experiments).
3. *In vivo* growth of MB49-I cells transduced with shDcn1, shDcn2 or Scr in C57Bl/6J female hosts. Mice were sacrificed 7 days after orthotopic injection and weight of individual bladders is shown (pool of 2 independent experiments). Horizontal bar = median. **: *p* = 0.0010; ***: *p* < 0.0001 (Mann–Whitney test).
4. Secretion level of decorin quantified by ELISA in the 24 h-conditioned medium of MB49 cells transfected with a decorin-expressing (MB49-Dcn), or a control plasmid (MB49-CMV). Bars represent mean ± SEM of duplicate wells from one representative experiment out of two. (n.d.: not detected).
5. *In vivo* growth of MB49-Dcn and MB49-CMV after subcutaneous injection in male mice. Growth in individual mice (middle and left panels) and tumour volume at day 20 (right panel) are shown (pool of 2 independent experiments). Horizontal bar = median. *: *p* = 0.0206 (Mann–Whitney test).
6. Blood vessels quantified by anti-CD31/Pecam1 immunohistochemistry in orthotopic MB49 and MB49-I tumours. Representative photographs (left panels, ×40) and quantification in 2–3 fields of 5–7 individual tumours (right panel) are shown. Stars indicate one blood vessel. ***: *p* < 0.0001 (Mann–Whitney test).
7. Expression of VE-Cadherin (*Cdh5*), VEGFR-2 (*Kdr*) and *Pecam1* in MB49-I-shDcn1, MB49-I-shDcn2 or MB49-I-Scr tumours were analysed by quantitative RT-PCR 20 days after tumour injection. A.U. of gene expression for 5–6 individual mice per group are shown.

Since decorin did not regulate MB49-I cells proliferation (Fig 5B), we hypothesized that it affects the tumour environment. Reduced expression of decorin in MB49-I-shDcn1 and MB49-I-shDcn2 did not modify the amount and relative proportions of immune cells infiltrating the tumour (Supporting Information Fig S4), thus suggesting that decorin does not affect directly anti-tumoural immune response. The extent of tumour angiogenesis is another striking difference between MB49 and MB49-I growing *in vivo*, as evidenced by the strong macroscopic haematuria induced by orthotopic MB49-I but not MB49 (Lodillinsky et al, 2009), and by the increased number of blood vessels present in MB49-I (Fig 5F). Since decorin has been shown to promote tube formation from endothelial cells and inflammation-induced angiogenesis (Fiedler & Eble, 2009; Nelimarkka et al, 2001; Schonherr et al, 2001; Schonherr et al, 1999; Schonherr et al, 2004), we next asked whether decorin plays a role in angiogenesis in our tumour model. Orthotopically growing MB49-I-Scr induced strong haematuria in 5/6 host mice after only 7 days after injection. In contrast, 1 out of 6 mice bearing MB49-I-shDcn2 and none of the mice bearing MB49-I-shDcn1 displayed haematuria. Furthermore, quantification of the expression of endothelial cell markers by qRT-PCR in subcutaneous tumours showed a slight decrease in the expression of VE-cadherin (*Cdh5*) in MB49-I-shDcn1 and MB49-I-shDcn2 tumours, and of VEGFR-2 (*Kdr*) and CD31 (*Pecam1*) in MB49-I-shDcn1, as compared to MB49-I-Scr tumours (Fig 5G). This observation suggests that decorin may promote MB49-I growth at least partly by promoting tumour angiogenesis.

### Decorin is overexpressed in muscle-invasive as compared to non-muscle-invasive human bladder carcinoma

**Figure 6:**
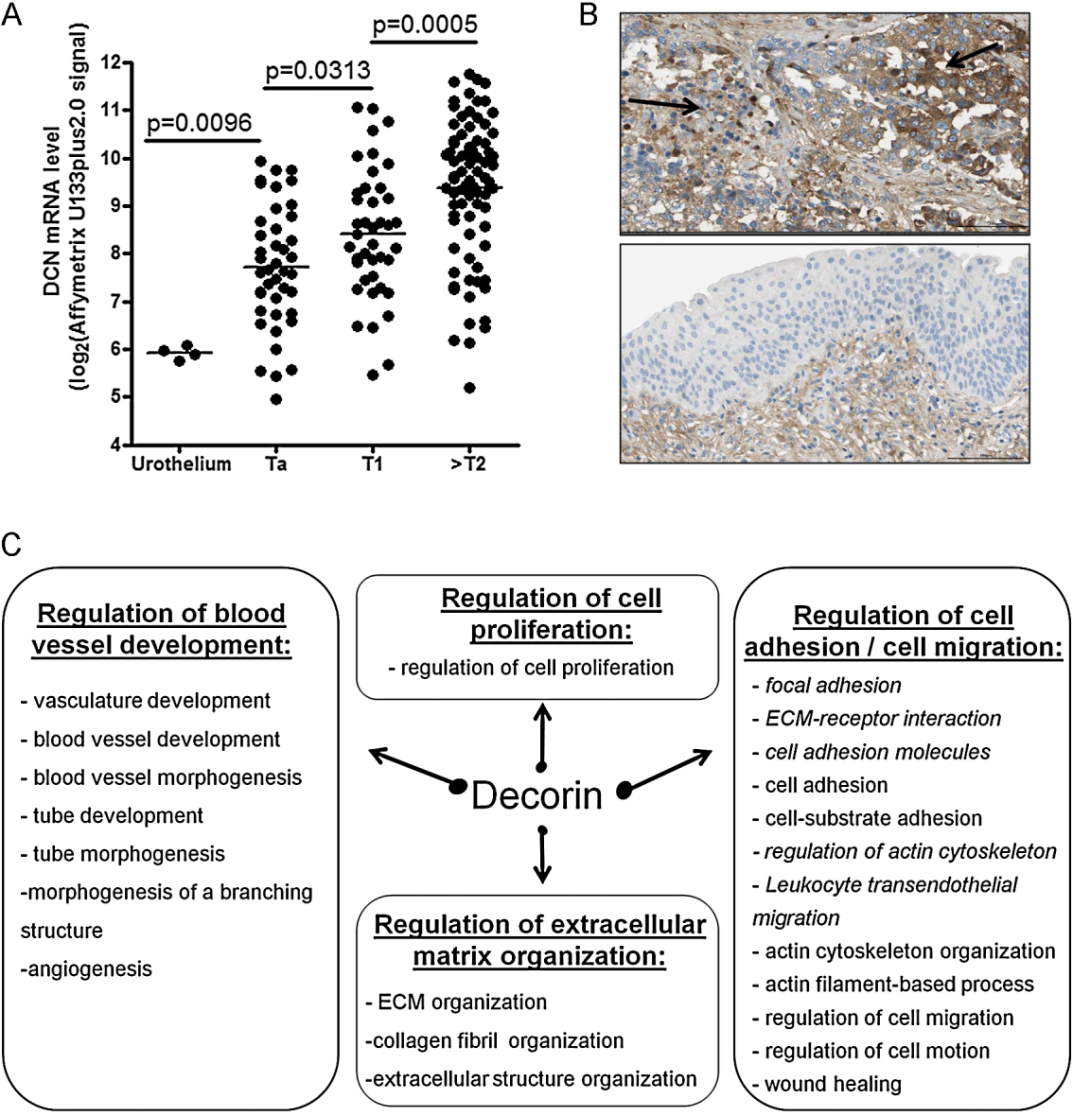
Decorin is overexpressed in human bladder tumours during progression and correlated with angiogenesis related-genes within MIBC

To determine whether decorin could also participate in development of human invasive bladder tumours, we next quantified decorin (*DCN*) mRNA expression level in our set of 162 tumour and 4 normal samples using Affymetrix U133 plus2.0 microarrays (Fig 6A). A significant overexpression of decorin was observed in the muscle-invasive bladder carcinoma (MIBC) (>pT2) as compared to superficial/non-muscle invasive bladder carcinoma (NMIBC) (pTa or pT1) and to normal urothelium (Fig 6A). These data were confirmed using another type of microarray (Affymetrix exon 1.0st) to analyse the same set of tumours (Supporting Information Fig S5A), but also by analysing publicly available transcriptomic datasets from three independent series of MIBC and NMIBC (Lindgren et al, 2010; Riester et al, 2012; Sanchez-Carbayo et al, 2006) (Supporting Information Fig S5B). In our dataset, there was no correlation between *DCN* expression and DNA copy number at the *DCN* locus, suggesting a transcriptional deregulation in tumours (Supporting Information Fig S6).

1. Decorin (*DCN*) mRNA expression in our set of 162 human bladder tumours. Expression levels in individual samples, and the median (horizontal bar) of each group of samples are represented. *p* was calculated by Wilcoxon's rank sum test. Urothelium = normal tissue; Ta, T1 = non-muscle-invasive bladder carcinoma (NMIBC); >pT2 = muscle-invasive bladder carcinoma (MIBC).
2. Expression of decorin (DCN) protein in a section of a high-grade invasive human tumour (upper panel, arrows indicate tumour area containing epithelial cells) and of a normal bladder (lower panel). Scale bar 100 µm. Pictures were extracted from the human protein atlas portal (http://www.proteinatlas.org/). Immunolabelling of decorin was performed using two different antibodies showing the same localization of decorin.
3. Pathways and gene ontology biological processes significantly enriched within genes positively correlated with *DCN* in human MIBC. Genes correlated with *DCN* expression in MIBC were determined using Pearson's correlation (*p* < 1.10^−6^ and *R* > 0.5) (Supporting Information Table S1). Significant enrichment of genes related to physiological pathways (italics) and gene ontology annotations relative to biological process within genes positively regulated with *DCN* were determined by DAVID software (corrected *p*-value < 0.01 and corrected *p*-value < 0.001, respectively) (Supporting Information Tables S1 and S2). All these pathways and gene ontology annotations, except one, can be grouped into four known functions of decorin.

DCN expression is mostly described as correlated to myofibroblast and fibroblast extent (Hocking et al, 1998). To determine if *DCN* overexpression observed in MIBC was only due to an increased infiltration of myofibroblasts within the tumour during tumour progression, we took advantage of the human protein atlas portal (http://www.proteinatlas.org/) (Uhlen et al, 2010). Immunohistochemistry showed a strong membranous and cytoplasmic expression of decorin by epithelial tumour cells together with a weaker expression by stromal cells (Fig 6B, upper panel) whereas normal urothelial cells did not express DCN (Fig 6B, lower panel) in agreement with our transcriptomic data for normal urothelium samples (Fig 6A).

To identify the physiological pathways that DCN could affect during bladder tumour progression, we identified genes with expression profiles correlated or anti-correlated with *DCN* within MIBC (Pearson correlation coefficient). Considering genes with absolute Pearson correlation coefficient (*R*) above 0.5 (*p*-value < 1.10^−6^), we identified only 15 anti-correlated genes and 471 genes positively correlated with *DCN* (Supporting Information Table S1). The latter genes were analysed further using DAVID tools (Functional Annotation Bioinformatics Microarray Analysis) (http://david.abcc.ncifcrf.gov) (Huang da et al, 2009a; Huang da et al, 2009b), to identify enrichment in Gene Ontology biological processes and in functional pathways (Supporting Information Table S2). Gene ontology biological process enrichment was also evaluated using the Gorilla application, which allows ‘tree’ visualization (Eden et al, 2007; Eden et al, 2009) (Supporting Information Fig S7). We observed enrichments in pathways and gene ontology annotations related to regulation of blood vessel development including angiogenesis (Fig 6C). Angiogenesis-related genes correlated with *DCN* expression in human MIBC included *K*DR (VEGF-R2), *CDH5* (VE-Cadherin) and several endothelial cell markers (PECAM1, VCAM1, CD34, CAM, JAM1, JAM2) (Supporting Information Tables S1 and S2). *K*dr and *Cdh5* were also downregulated in mouse MB49-I tumours following decorin knockdown (Fig 5G), thus suggesting involvement of DCN in regulation of angiogenesis during human bladder tumour progression. In addition, several genes related to cell migration, adhesion to substrate and regulation of ECM organization were also co-regulated with *DCN* (Fig 6C), suggesting another link of decorin with tumour cell migration or invasion.

### Decorin promotes invasiveness of human bladder tumour cells

**Figure 7:**
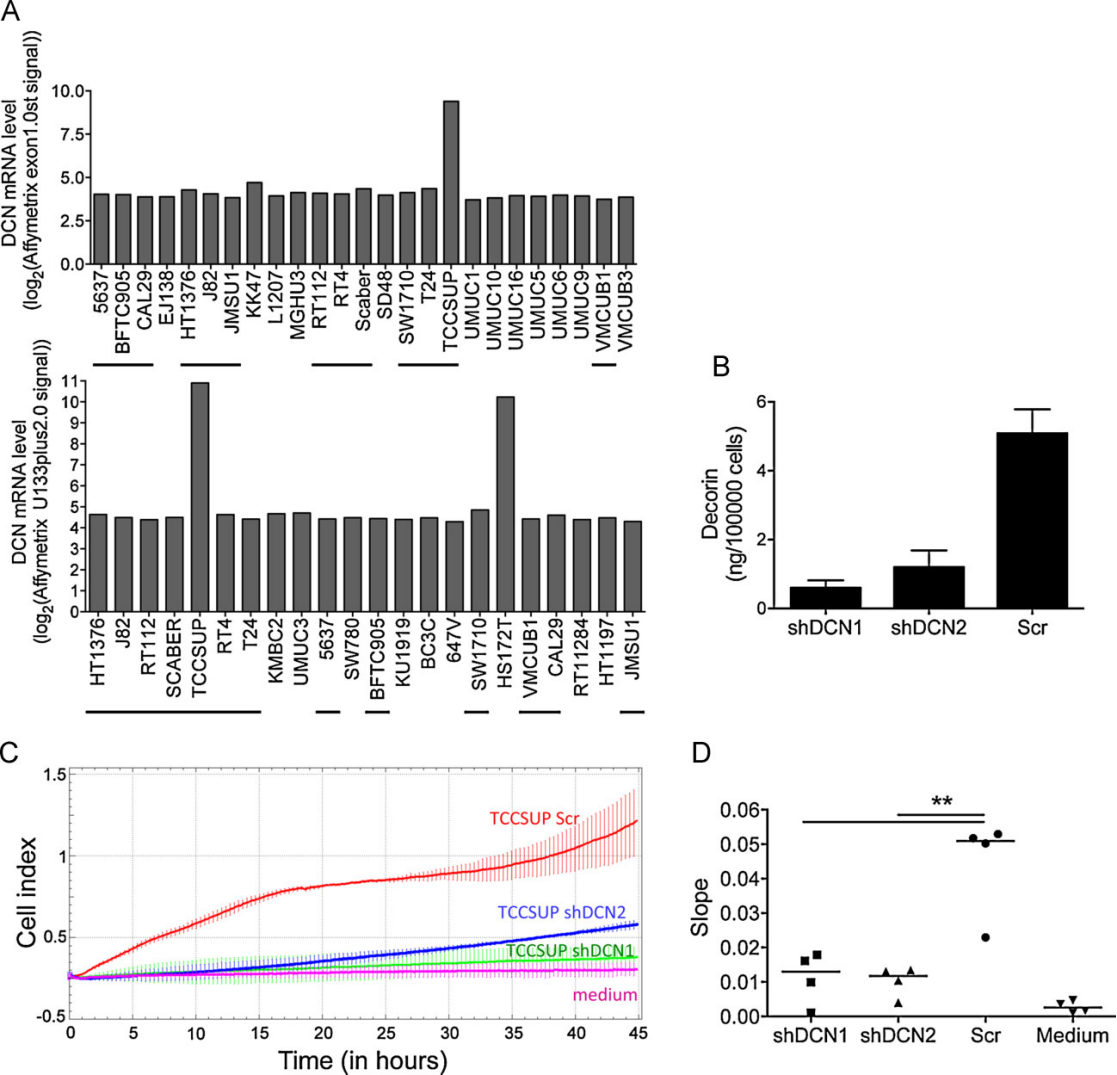
DCN promotes invasiveness of a human bladder tumour-derived cell line

To determine whether DCN could play similar pro-tumoural functions in human bladder tumours as in the MB49-I mouse model, we first analysed expression of *DCN* mRNA in human bladder carcinoma cell lines. Out of 34 cell lines for which transcriptomic data were available (from our own dataset: 25 cell lines upper panel, or from publicly available data: 22 cell lines lower panel, 13 cell lines in common; Fig 7A), we identified two with strong expression of the *DCN* mRNA, TCCSUP and HS172T. Lentiviruses expressing two different shRNA specific for the human gene were used to knock-down DCN expression in the TCCSUP cell line, leading to more than 80% reduction in secretion of the protein *in vitro* (Fig 7B). As observed with the MB49-I cells, *DCN* knockdown did not alter TCCSUP growth rate *in vitro* (Supporting Information Fig S8A). TCCSUP cells did not form tumours when injected subcutaneously in immunodeficient mice (up to 10^7^ cells/flank without or with matrigel, in nude or scid mice) so we could not address the pro-tumoural role of DCN *in vivo* in this model. However, the study of genes correlated with DCN in MIBC suggested a pro-invasive effect of decorin, which we therefore investigated *in vitro*. In an invasion assay using the xCELLigence real-time measurement device (Fig 7C, D), we observed a significant impairment of migration of shDCN-expressing TCCSUP through matrigel to the lower compartment of the Boyden chamber, as compared to the control shScr-expressing cells. This pro-invasive effect of decorin was similarly observed in the MB49/MB49-I model: decorin overexpression increased invasiveness of MB49 (Supporting Information Fig S8B), whereas decorin silencing reduced invasiveness of MB49-I (Supporting Information Fig S8C) in the *in vitro* matrigel invasion assay. Altogether, our results show that decorin expression in human bladder tumours promotes progression of these tumours to an invasive stage.

A. Analysis of *DCN* mRNA expression levels in human bladder carcinoma-derived cell lines, from our own data set: 25 cell lines (upper panel), or from public results from the Broad-Novartis Cancer Cell Line Encyclopedia: 22 cell lines (lower panel). The 13 common cell lines are underlined. Only TCCSUP and HS172T present high *DCN* mRNA levels.
B. Secretion levels of decorin quantified by ELISA in the 24 h-conditioned medium of TCCSUP infected with lentiviruses expressing two different shRNA to decorin (shDCN1 and shDCN2) or a control shRNA (Scr). Mean ± SEM of 2 independent experiments is shown.
C,D. Quantification of matrigel invasion by TCCSUP using the xCELLigence device. Cells were seeded in the upper chamber containing matrigel of CIM-plates. (C) Example of cell index (CI, *y*-axis) as a function of time (*x*-axis), for TCCSUP expressing (shScr) or not (shDCN1, shDCN2) decorin, as compared to the signal obtained in the absence of cells (medium). (D) Quantification of slope values at 24 h in 4 wells from 2 independent experiments. **: *p* = 0.003 for shDCN1 and shDCN2 compared to Scr, 2-way ANOVA, Tukey's post-hoc test.

## DISCUSSION

Effective anti-tumour immune response requires appropriate recruitment and activation of immune cells. However, despite expression of tumour antigens in most tumours, including bladder tumours (Nishiyama et al, 2001; Sharma et al, 2003), escape from T-cell immunity is more often the rule rather than an exception. In this study, we examined and compared the immune response going on during development of bladder tumours induced by two cell lines: MB49 and its more invasive variant MB49-I. Our analysis of adaptive immune response during MB49 and MB49-I development and exploration of molecules secreted by both tumours showed that MB49-I has developed two mechanisms to acquire invasion properties: loss of putative tumour antigens and overexpression of decorin.

The expression of the male transplantation H-Y antigens by MB49 was shown to induce an anti-tumour immune response when injected in female mice (Halak et al, 1999). Our results showed that, in stark contrast to MB49, MB49-I has lost the Y chromosome and fails to induce any proliferation of anti-H-Y T lymphocytes. Given that MB49-I had been obtained by successive passages *in vivo* in male animals, there was no particular reason to think that male antigens would have been selectively eliminated. Indeed, our experiment showing that the differential sensitivity to immune system of MB49 and MB49-I is also observed in male hosts, which are tolerant to H-Y antigens (Fig 3E), suggest that a large set of tumour antigens have been lost by MB49-I. Since recent data from Next Generation Sequencing (Gui et al, 2011) show that bladder carcinomas contain numerous non-silent mutations, and thus potential neo-antigens, our demonstration of a role of adaptive immune responses to tumour neo-antigens in the mouse model of bladder cancer is relevant to the human setting.

In addition, our results first pointed out a defect in CD8^+^ T lymphocytes infiltration and an increase in Treg cells in MB49-I immune infiltrates. These data suggested that MB49-I induced an immunosuppressive environment that allowed its more invasive phenotype. Indeed, earlier studies have shown that MB49 cell can promote IL-10 production by host splenocytes and IL-10^−/−^ mice displayed prolonged survival and increased capacities to reject tumour after MB49 injection (Halak et al, 1999). However, flow cytometry analysis of cytokines produced by infiltrating CD4^+^ and CD8^+^ T cells did not show increased percentage of IL-10-producing cells in MB49-I tumours, ruling out IL-10-dependent tolerance as a major cause for increased invasiveness of MB49-I tumours. In contrast, we found high frequencies of IL-17A-producing T (Th17 and Tc17) cells in MB49-I tumours as compared to MB49 ones. A large body of literature demonstrates that Th17 cells and their hallmark cytokines can have opposite roles in modulating growth in different tumour systems (Maniati et al, 2010). Results from two studies in prostate and ovarian cancer patients have suggested both beneficial and detrimental implications of Th17 cells in tumour development (Kryczek et al, 2007; Sfanos et al, 2008). Our observation thus suggests that in this model of invasive bladder tumour, IL-17 probably has a pro-tumoural effect.

The second physiological mechanism used by MB49-I to overgrow is independent of the adaptive immune system. Its existence was evidenced by the faster progression of MB49-I as compared to MB49, in immunodeficient hosts (Fig 3D). This observation prompted us to compare the secretome of both cells, and we thus identified decorin as a major difference between the two tumours. Our results demonstrate that decorin is required and sufficient to promote progression of the MB49 bladder tumour *in vivo* (Fig 5). Several studies have demonstrated that *de novo* decorin expression in tumour cell lines can inhibit their proliferation *in vitro* (De Luca et al, 1996; Iozzo et al, 1999; Nash et al, 1999). However, decorin is endogenously expressed by tumour cells and is not anti-proliferative in our model since *in vitro* growth of MB49-I was not increased by inhibition of decorin. We thus hypothesized that MB49-I-derived decorin acts by interacting with other cells in the tumour microenvironment, but we had not observed obvious differences in the immune microenvironment generated by decorin-impaired MB49-I cells. Given the previously described promoting role of decorin on endothelial cells and on angiogenesis in cornea (Fiedler & Eble, 2009; Nelimarkka et al, 2001; Schonherr et al, 1999; Schonherr et al, 2001; Schonherr et al, 2004), we tested whether decorin produced by MB49-I cells would favour angiogenesis within the tumour bed. Our results suggest that MB49-I cells developed a more aggressive phenotype during *in vivo* passage in part through acquisition of decorin expression that allowed enhanced angiogenesis. In addition, we also demonstrated a role for decorin in promoting invasiveness into matrigel of the mouse tumour cell line *in vitro* (Supporting Information Fig S8B and C). Of note, our secretome analysis also confirmed that MB49-I secretes higher level of the metalloproteinase MMP9 than MB49 (Fig 4A), which, as described previously (Lodillinsky et al, 2009), increases ability to invade the surrounding tissue and also induces an angiogenic switch promoting invasion. It would now be interesting to check whether decorin regulates MMP9 expression in MB49-I cells, as demonstrated in oral squamous carcinoma cell lines (Dil & Banerjee, 2011; Dil & Banerjee, 2012).

Decorin has been found to have a tumour suppressor role in several studies (Csordas et al, 2000; Iozzo et al, 1999; Santra et al, 2000), but other works (Benet et al, 2012; Dil & Banerjee, 2011; Zafiropoulos et al, 2008) and ours correlate decorin with increased tumour invasion and metastasis. These opposite role could be due to different decorin isoforms and/or protein localization (Dil & Banerjee, 2012). Lumican, another small leucine proteoglycan, has also been demonstrated to display dual anti- or pro-tumoural activity depending of tumour types (Theocharis et al, 2010) and is highly correlated to decorin in human MIBC (*R* = 0.9) (Supporting Information Table S1). It would be interesting to determine if, like decorin, lumican displays pro-tumoural activities in our MB49-I model.

Although obtained with a particular model of mouse bladder tumour, we show here that our results are relevant to human bladder cancers. In several libraries of human bladder tumours, we observed that decorin was upregulated in muscle-invasive as compared to non-invasive bladder tumours (Fig 6A and Supporting Information Fig S5). In addition, in our data sets, *DCN* was overexpressed in the tumoural as compared to healthy urothelium. Analysis of the protein by immunohistochemistry obtained from the human protein atlas portal confirmed overexpression of decorin in epithelial tumour cells as compared to normal epithelial cells. However, in normal bladder, decorin was observed in stroma and in particular in muscle cells, in which we also detected strong decorin mRNA expression (Supporting Information Fig S9). This observation could explain the discrepancy between our study and a previous report showing downregulation of *DCN* expression in human bladder carcinoma (Iozzo et al, 2011). In this other study, bladder tumours were compared to whole bladder or bladder mucosa, which contain stroma and smooth muscle cells from the bladder wall in addition to urothelium. Of note, however, when comparing muscle-invasive to superficial tumours, the authors observed, like us, overexpression of decorin in MIBC. Interestingly, using our exon array data for bladder wall muscle, normal urothelium and bladder tumours, we observed that *DCN* was transcribed from at least two promoter regions in bladder tissues. In muscle tissues, *DCN* appears to be preferentially transcribed from a promoter that is not expressed in normal and tumoural tissues. Although these different promoters induce expression of the same protein, their different use in the two tissues allows us to rule out that decorin overexpression in tumours is due to muscle infiltration (Supporting Information Fig S9). Another study has also described stage-related accumulation of decorin during progression of laryngeal and pancreatic cancer, also associated with tumour-specific post-translational modifications (Skandalis et al, 2006a; Skandalis et al, 2006b). Notably, when analysing isolated tumour cell lines, we observed that only a small proportion of them (2/34) actually express decorin (Fig 7A), suggesting that the tumour microenvironment promotes decorin expression by the tumour cells *in situ*, or, alternatively, that part of the expression of decorin in patients biopsies could come from activated myofibroblasts surrounding the tumour cells.

One of the few examples of successful immunotherapy is the treatment of NMIBC by BCG. However, in MIBC, BCG is not effective and surgery, chemotherapy and radiotherapy are the main treatments used in these settings. Our observation that tumour antigens are lost during progression of bladder tumours could explain, at least partly, the failure of efficient induction of anti-tumour immune responses by BCG in MIBC. Finally, our data demonstrate that decorin is associated with progression and promotes invasiveness in human bladder cancer, as proposed from our analysis of a mouse invasive bladder tumour cell variant. Decorin may thus offer the potential for therapeutic exploitation in advanced bladder cancer.

## MATERIALS AND METHODS

### Mice

C57Bl/6J mice were obtained from Charles Rivers Laboratories (L'Arbresle, France). CD45.1^+^ C57Bl/6J, *Rag2*^*−/−*^ and *Rag2*^*−/−*^ transgenic mice containing monoclonal T lymphocytes specific for the male antigen H-Y, either as a Dby-derived peptide associated with MHC class II IA^b^ [Marilyn (Lantz et al, 2000)] or as a Uty-derived peptide associated with MHC class I D^b^ [MataHari (Valujskikh et al, 2002)], were bred in SPF conditions at CERFE (Centre d'Exploitation et de Recherche Fonctionnelle Expérimentale, Evry, France). Experiments were performed with approval of the Ile-de-France ethical committee for animal experimentation.

### Tumour cell lines

The parental murine bladder carcinoma cell line MB49 and the invasive variant MB49-I have been described before (Lodillinsky et al, 2009). TCCSUP was purchased from DSMZ. All cell lines were cultured in DMEM supplemented with glutamax, 10% FCS, 100 U/ml penicillin and 100 µg/ml streptomycin (Invitrogen). Luciferase-expressing MB49 and MB49-I were obtained by stable transfection with the pGL4.50 luc2/CMV/Hygro plasmid (Promega), and selection of hygromycin-resistant subclones displaying similar luciferase activity *in vitro* was analysed by a Berthold Centro LB 960 luminometer.

### *In vivo* tumour implantation

For orthotopic tumour implantation, female mice were anesthetized with 150 µl of a Ketamine/Xylasine solution (15 and 2%, respectively) and a 24-gauge Teflon catheter was introduced into the bladder lumen through the urethra. To prepare the bladder for tumour implantation, a point lesion was induced in the bladder wall by an electrocautery unit (Micromed MD1; level 3; 2 pulses per mouse) as already described (Dobek & Godbey, 2011). Then, 1 × 10^5^ of either MB49 or MB49-I cells in 100 µl of PBS were instilled into the bladder. When indicated, control mice were electrocauterized and PBS was instilled into the bladder. Syringes containing the cell suspension were maintained into the catheter for 20 min to allow cell attachment to the bladder wall.

For heterotopic tumour growth, 1 × 10^5^ MB49 or MB49-I cells in 100 µl of PBS were injected subcutaneously in the flank of syngeneic hosts, and tumour growth was measured with a caliper twice weekly. Tumour volume was expressed as length × width × [(length + width)/2]).

### Bioluminescence imaging

The growth of orthotopically implanted luciferase-expressing MB49 and MB49-I tumours was monitored with IVIS Lumina II (Caliper Life Sciences). Ten minutes before imaging, mice previously shaved in the abdominal area were anesthetized and given luciferin i.p. (150 mg/kg, Caliper).

### Flow cytometry

Ten to 12 days after tumour implantation, mice were sacrificed by cervical dislocation and bladders were harvested. Single cell suspension were prepared after Collagenase/DNAse digestion at 37°C during 30 min. Cells were pre-incubated with 2.4G2 mAb to block non-specific binding to Fc receptors and then stained with the following mAbs: CD45.2 (FITC; 104), CD4 (Pe-Cy7; RM4-5), CD8 (Pacific blue; 53-6.7), TCRβ (PerCP-Cy5.5; H57-597), CD19 (APC-Cy7; 1D3), NKp46 (PE; 29A1.4), Foxp3 (APC; FJK-16s), IL-17A (PE; TC11-18H10), IFN-γ (APC; XMG1.2), IL-10 (PE; JES5-16E3).

For intracellular cytokine staining, cells were restimulated with phorbol myristate acetate (50 ng/ml, Sigma) and ionomycin (500 ng/ml, Sigma) for 4 h in the presence of GolgiPlug (BD Biosciences). After cell surface staining, intracellular staining was performed with the eBiosciences kit and protocol for Foxp3 (Foxp3 staining kit; eBiosciences) and cytokine staining was done according to manufacturer's instructions with Fix & Perm reagents (Caltag Laboratories). Cells were acquired on a MacsQuant cytometer (Miltenyi Biotec) and analysed with FlowJo software (Treestar).

### Analysis of T-cell response *in vivo*

Female CD45.1^+^ C57Bl/6J mice were injected in the footpad with 3 × 10^5^ MB49 or MB49-I cells. The next day, splenocytes (4.10^6^ mouse^−1^) from CD45.2^+^ MataHari and Marilyn mice stained with CellTrace Violet Cell Proliferation Kit (Life Technologies) were injected intravenously. Mice were sacrificed 5 days later and popliteal lymph nodes cells were analysed by flow cytometry for the presence of dividing CD45.2^+^ Vβ6^+^/CD4^+^ or Vβ8^+^/CD8^+^ T cells.

### Analysis of H-Y antigen expression and Y chromosome presence

RNA was extracted from MB49, MB49-I and male splenocytes using Qiagen Rneasy kit (Qiagen) and reverse-transcription were performed on 1 µg of RNA with AMV-RT (Finnzyme, Esposo, Finland). Expression of the two H-Y antigens Uty and Dby was measured by quantitative RT-PCR on a Lightcycler LC480 (Roche) using Taqman primer pairs and probes for quantitative real-time PCR from Applied Biosystems (Genbank accession numbers in parentheses; assay identification numbers from Applied Biosystems provided here): Uty (NM_009484.2), Mm00447710_m1; Dby (NM_012008.2), Mm00465349_m1; Gadph (NM_008084.2), Mm99999915_g1. Cycle threshold (Ct) for mouse *Uty* and *Dby* were normalized to Ct for *Gapdh* and results were expressed as arbitrary units (AU: 2^Ct(Gapdh)-Ct(gene)^ × 1000). Presence of the mouse Y chromosome was analysed by DNA FISH according to manufacturer's protocol (Metasystem, Germany), and fluorescent cells were visualized using a Leica DM4000 microscope.

### Measurement of cytokine and chemokine release using antibody arrays and ELISA

RayBio® Mouse Cytokine Array C2000 (RayBiotech Inc., Norcross, GA) allowing simultaneous analysis of 144 different cytokines on 3 antibody arrays was used. One millilitre of 48 h conditioned medium from 3 × 10^5^ MB49 or MB49-I cells (or of unconditioned medium) was added to each membrane and analysed according to manufacturer instructions. For ELISA experiments, 24 h conditioned medium from 2 × 10^5^ cells were used. Duoset ELISA (R&D Systems) reagents were used for quantification of indicated cytokines in conditioned mediums obtained in the same conditions described above.

### Modification of Decorin expression in tumour cell lines

Decorin overexpression in MB49 cells was achieved using the pCMV6-Entry plasmid containing cDNA of mouse Decorin (NM_007833) purchased from Origene (Rockville, MD, USA). Transfected MB49 cells were selected with geneticin (1.5 mg/ml). Knock-down of decorin expression in MB49-I or TCCSUP, was performed using shRNA purchased from Sigma, expressed in lentiviruses produced from pLKO.1 plasmids, as described (Moffat et al, 2006). A control lentivirus termed Scr, expressing a scrambled sequence of shRNA specific for a non-murine gene (GFP), was used in parallel with gene-specific lentiviruses. Lentivirus-infected cells were selected with puromycin (2 µg/ml)), and specific knock-down of the gene of interest was evaluated by measuring secretion of decorin in 24 h-conditionned medium.

Sequences of the two shRNA to mouse decorin, recognizing all isoforms of the mRNA, were:

shDcn1: CGGG**CCTGAAAGGACTGATTAATT**CTCGAG**AATTAATCAGTCCTTTCAGG**CTTTTTG
shDcn2: CCGGG**TCCGGTATTGGGAAATCTTT**CTCGAG**AAAGATTTCCCAATACCGGA**CTTTTTG

Sequences of the two shRNA to human decorin, recognizing all isoforms of the mRNA, were:

shDcn1: CCGG**CCGTTTCAACAGAGAGGCTTA**CTCGAG**TAAGCCTCTCTGTTGAAACGG**TTTTTG
shDcn2: CCGG**CGACTTTATCTGTCCAAGAAT**CTCGAG**ATTCTTGGACAGATAAAGTCG**TTTTTG

### Immunohistochemistry for endothelial cells in mouse tumours

Bladders bearing MB49 and MB49-I tumours were harvested at d15 and fixed in formalin as described before (Lodillinsky et al, 2009), before paraffine inclusion and staining with anti-CD31/Pecam1 antibody (M-20 sc-1506, Santa Cruz). CD31+ blood vessels were manually counted under ×40 magnification in 2–3 sections of 5–7 tumours each.

### Quantitative RT-PCR analysis of angiogenic markers in bladder tumours

Mice bearing MB49-I-shDcn1, MB49-I-shDcn2 or MB49-I-Scr were sacrificed 20 days after subcutaneous injection and tumours were harvested. After tissue lysis using FastPrep^R^-24 (MP biomedicals), RNA was extracted with Qiagen Rneasy kit (Qiagen) and reverse-transcription was performed as described above. Taqman probes and primer pairs for *Gapdh*, VE-Cadherin (*Cdh5*), VEGFR-2 (*Kdr*) and CD31 (*Pecam1*) were purchased from Applied Biosystems. Cycle threshold (Ct) method was used to quantify gene expression, as compared to *Gapdh*.

### Analyses of human bladder samples and Affymetrix microarray data

A set of human bladder carcinomas was collected from patients treated surgically between 1988 and 2006 at Henri Mondor Hospital (Créteil, France), Institut Gustave Roussy (Villejuif, France) and Foch Hospital (Suresnes, France). All tumours were pathologically reviewed, staged according to the 2009 TNM. All patients provided written informed consent and the study was approved by the ethics committees of the various hospitals. Normal urothelial samples were obtained from organ donors (Diez de Medina et al, 1997), Expression of desmin, a specific smooth muscle cell marker, was measured by RT-qPCR, and tumour samples with expression higher than 25% of the median of 5 normal bladder smooth muscle were considered over-contaminated with stroma, and thus not included in the analysis. Human bladder samples were analysed with Affymetrix HG-U133 Plus 2.0. Data for *DCN* and *DCN*-correlated genes among MIBC (pearson *R* > 0.5) are available for 162 tumours and 4 normal urothelium (Supporting Information Table S3). The microarray data described here are available from ArrayExpress (http://www.ebi.ac.uk/arrayexpress/) under the accession numbers E-MTAB-1803 for the muscle invasive bladder tumours and E-MTAB-1940 for the non-muscle invasive tumours and normal samples.

Twenty-five bladder cancer derived cell lines were obtained from ATCC or DSMZ. RNA, DNA and proteins were extracted from frozen human bladder samples and from human bladder cancer cell lines grown to 70% confluence, by centrifugation through a cesium chloride density gradient. Cell lines were analysed with Affymetrix Exon1.0st arrays and *DCN* mRNA levels were extracted from these data (Fig 7A).

### Publicly available gene expression datasets

Three independent datasets of human bladder tumours were used: (Riester et al, 2012) (GSE31684) containing data obtained using Affymetrix HG-U133 Plus 2.0 array for 93 bladder tumours, (Lindgren et al, 2010) (GSE19915) containing data obtained from cDNA microarray for 144 bladder tumours, and (Sanchez-Carbayo et al, 2006) containing data obtained using Affymetrix U133A array for 105 bladder tumours. Gene expressions (Affymetrix U133 plus 2.0) for 22 bladder cancer-derived cell lines were downloaded from the Broad-Novartis Cancer Cell Line Encyclopedia (CCLE, http://www.broadinstitute.org/ccle).

### Matrigel invasion assay

The xCELLigence real-time impedance measurement system (ACEA Biosciences) was used with CIM tissue culture microplates, *i.e*. Boyden chamber-type tissue culture wells, with lower and upper compartments separated by an 8 µm pore-containing membrane. The impedance-measuring electrodes are present on the lower side of the pore-containing membrane and allow detection of cells, which have migrated through the pores. The bottom chamber of CIM-Plate wells were loaded with 160 µl medium containing 1% FCS (MB49, MB49-I) or 10% FCS (TCCSUP), the transwell insert was positioned and 25 µl/well of matrigel (BD Pharmingen) diluted 1/10 in medium without FCS was seeded in the upper chamber. CIM-Plates were transferred onto the xCELLigence instrument and left to equilibrate for 2 h, at which time background impedance was measured (= t0). Plates were taken out and 75 µl of medium-1% FCS containing 4.10^4^ cells were loaded in each well. Plates were placed back in the xCELLigence device, which was set to measure impedance every 5 min for the next 72 h. Data were plotted using the RTCA software as cell index (CI) value over time, and the slope of CI between 0 and 24 h (TCCSUP) or 0 and 36 h (MB49/MB49-I) was chosen as the shortest time point showing high enough CI.

### Statistical analyses

Experiments were performed in mice at least 2 and up to 7 times, amounting to between 4 and up to 31 individual samples. In human studies, a total of 162 individual tumours were analysed. In most figures, results are represented as individual dots corresponding to individual samples.

Mann–Whitney test was used to compare tumour growth, percentages of immune cells in groups of mouse tumours and quantitative RT-PCR data, using Prism 4.0 software. *p*-values less than 0.05 were considered statistically different.

Genes co-expressed with *DCN* were identified by pairwise correlation analysis between expression profiles of *DCN* and each other gene measured by the Affymetrix microarrays, using the Pearson's rank correlation coefficient. Genes showing significant correlation value (*p* < 1.10^−6^) greater than 0.5 or below −0.5 in the set of muscle invasive tumours (>T2) are listed in Supporting Information Table S1.

For the xCELLigence-based invasion assay, results from individual wells in 2–4 experiments were used and statistical analysis was performed as described (Tibaldi et al, 2013) by 2-way ANOVA followed by Tukey's post-Hoc test, with *p*-values adjusted for multiple testing, using the R software (version 2.13.1, http://www.R-project.org/). The significance level was set at 5%.

### The paper explained

PROBLEM

Bladder cancer is the fourth type of cancer in men in developed countries, and the fifth cause of death from cancer. Non-invasive tumours are sensitive to immunotherapeutic treatment using intravesical instillations of bacillus Calmette-Guerin (BCG), but 30–50% of treated patients relapse and develop metastases for which no effective therapy exists. On the other hand, muscle-invasive tumours are not sensitive to BCG therapy, and bladder excision is the only treatment. It is therefore urgent to understand the molecular mechanisms of progression of invasive bladder tumours, in order to develop alternative therapies to BCG.

RESULTS:

By analysing *in vivo* the murine bladder tumour cell line MB49, and its more invasive variant MB49-I, we show that the invasive tumour has become insensitive to destruction by the adaptive immune system, thus escaping this first means of control by the host. In addition, MB49-I has developed the ability to secrete decorin, which promotes angiogenesis and invasiveness and is necessary and sufficient for *in vivo* progression of this tumour. Similarly in human patients, we demonstrate a significant overexpression of decorin in muscle-invasive bladder cancers, with a correlative increase in expression of angiogenesis-specific genes, and a crucial role to promote invasive ability of the tumour cells. These observations are unexpected, since decorin had so far mainly been shown to be anti-tumoural in various types of cancers.

IMPACT:

Our data demonstrate a role of the immune system in controlling a poorly invasive bladder tumour, probably explaining the efficacy of BCG therapy in human. More strikingly, they highlight a pro-tumoural role of decorin in invasive bladder cancer, and thus suggest the use of decorin as a therapeutic target in muscle-invasive bladder cancer.

## Author contributions

MEB, IBP, CT designed experiments, analysed data, wrote the article. MEB, SK, CL, GS, LT performed experiments and analysed data. EC, AK, ADR analysed data. TL, AL provided materials and participated in data analyses. YA, FR, OL, AME provided materials, participated in data analysis, revised the article.

## For more information

1. Websites used in this study include:
2. http://www.proteinatlas.org/
3. http://www.broadinstitute.org/ccle/home
4. https://tcga-data.nci.nih.gov/tcga/
5. http://david.abcc.ncifcrf.gov
