# Supplementary material for: An essential role for decorin in bladder cancer invasiveness
Source: EMBO Mol Med. 2013 Oct 20;5(12):1835–51. doi: 10.1002/emmm.201302655 (PMC3914526; doi:10.1002/emmm.201302655)
Supplement: Supplementary file 1 [file emmm0005-1835-sd1.pdf]

EMBO Molecular Medicine

An essential role for decorin in bladder cancer invasiveness

Mohamed El Behi, Sophie Krumeich, Catalina Lodillinsky, Aurélie Kamoun, Lorenzo Tibaldi, Gaël Sugano, Aurélien De Reynies, Elodie Chapeaublanc, Agnès Laplanche, Thierry Lebret, Yves Allory, François Radvanyi, Olivier Lantz, Ana María Eiján, Isabelle Bernard-Pierrot, Clotilde Théry

Corresponding author: Clotilde Théry, Institut Curie

Review timeline:

Submission date:	19 February 2013
Editorial Decision:	16 April 2013
Additional author correspondence:	21 June 2013
Additional editorial correspondence:	27 June 2013
Revision received:	15 July 2013
Editorial Decision:	09 August 2013
Additional Editorial Correspondence:	14 August 2013
Revision received:	06 September 2013
Accepted:	06 September 2013

Transaction Report:

(Note: With the exception of the correction of typographical or spelling errors that could be a source of ambiguity, letters and reports are not edited. The original formatting of letters and referee reports may not be reflected in this compilation.)

Editor: Céline Carret

1st Editorial Decision 16 April 2013

Thank you for the submission of your manuscript to EMBO Molecular Medicine. We have now heard back from the two referees whom we asked to evaluate your manuscript. I am sorry that it has taken so long to get back to you on your manuscript. While reviewers 1 and 2 delivered their evaluations in a timely manner, till to this date we did not receive the other reviewer's input.

As you will see from the reports below, both reviewers find the study to be of potential interest, however, they also raise a number of concerns that need to be addressed in a major revision of the manuscript.

while referee 1 is concise and would like to see the data backed up by another isogenic set of cell lines, referee 2 only suggests rewriting in places to help understanding the data and the experiments performed better. Moreover, while Referee 1 feels that the H-Y part of the study could be reduced, Referee 2 feels that this part would benefit from a much greater explanation and to our understanding this would be preferable and indeed improve the manuscript. Please see the last comment of Referee #2. Indeed our guidelines recommend to not use "data not shown", kindly address this issue as recommended.

Given these evaluations, I would like to give you the opportunity to revise your manuscript, with the understanding that the referee concerns must be fully addressed in the next final version of the manuscript. Please note that it is EMBO Molecular Medicine policy to allow a single round of revision in order to avoid the delayed publication of research findings. Consequently, acceptance or

rejection of the manuscript will depend on the completeness of your responses included in the next version of the manuscript.

EMBO Molecular Medicine has a "scooping protection" policy, whereby similar findings that are published by others during review or revision are not a criterion for rejection. Should you decide to submit a revised version, I do ask that you get in touch after three months if you have not completed it, to update us on the status.

Please also contact us as soon as possible if similar work is published elsewhere. If other work is published we may not be able to extend the revision period beyond three months.

I look forward to receiving your revised manuscript.

***** Reviewer's comments *****

Referee #1 (Comments on Novelty/Model System):

The long part of the manuscript on male proteins eliciting an immune response in a female mouse is irrelevan to the clinical setting.

Referee #1 (Remarks):

This paper describes an aggressive variant of a urothelial cancer cell line. The experiments are well selected and carried out in a professional manner.
However, I have reservations regarding two major points:
1. The immune response in females against male surface antigens is only of academic interest and not relevant t a clinical setting. this part of the paper could be reduced dramatically.
2. The findings on Decorin are interesting, but these findings should be repeated in another isogenic set of cell lines one being aggresive, the other indolent. It is not enought to show similar proteins in humans, the mechanism should be documented in at least two independent cell lines.

Referee #2 (Comments on Novelty/Model System):

The functional data that prove the key finding are generated in an murine system with an artificially implanted cell-line-derived tumor. The data are backed up by a descriptive analysis with corresponding human material, but as the key finding is very novel is can not yet be said, whether the murine system is suitable

Referee #2 (Remarks):

The manuscript by Mohamed El Behi et al. examines why the aggressive bladder carcinoma line MB49-I and its less aggressive parental line are different in their invasiveness. They pin it down to the expression of certain antigens (esp. H-Y) but, more importantly, to the expression of the proteoglycan decorin by the more aggressive variant. This is the key finding of this paper.

The key finding that decorin is sufficient and necessary for the observed enhanced tumor growth in vivo is new and interesting, because decorin-expression so far has been associated with reduced malignancy. The experiments performed to show this are well designed, appropriate controls and statistical analysis were performed. Although an artificial murine model system was used, the relevance for human bladder carcinoma is indicated by a correlative analysis of human patient material.
Unfortunately, the way parts of the manuscript (esp. at the beginning) are written does not comply to

the good experimental work of the authors. Some experiments are difficult to understand for anyone, who did not perform them, and the introduction contains information completely irrelevant to understand the paper, but lacks other necessary explanations.

Hence I conclude that a revised manuscript would be well worth publishing in EMBO Molecular Medicine, and that no additional experiments are needed, but that some parts of the manuscript must be rewritten to be more accurate and to make the work better understandable for the general readership.

Precise points:
1) The introduction does not say anything about decorin, although it is the key molecule of the manuscript. The function and role of decorin must be covered in the introduction.
BCG has nothing to do with the described experiments, so it should not be covered in that detail. The last paragraph of the introduction is in principle another abstract and hence redundant. If space is required, it can be omitted.

2) The explanation of figure 2 in the result section (page 7) in insufficient. What does the first panel (CD45.2 of CD45) tell? Did the authors stain for CD4/FoxP3 double positive or just for FoxP3 (it is differently described in the text and the figure). Since the MB49-tumors are already regressing while the MB49-I are growing, the observed differences may simply show the infiltration into a regressing vs. a growing tumor. The whole paragraph should be rewritten. The figure legend of figure 2 does not explain the figure properly, and should be more detailed.

3) On page 8, result section, it is implied (even though it is not explicitly stated) that H-Y was lost due to immune surveillance pressure during successive passages, however, as described later, this was done in male mice. The tumors ability to activate CD4 and CD8 T cells was examined with the Marilyn and MataHari system, which only detect H-Y-antigens (and should be briefly explained here). These were lost independently of immunological pressure, so the authors can not conclude that the tumor lost its general ability to stimulate T cells. They can only conclude, from the differences observed in the RAG KO mice, that the adaptive immune system does, or does not play a role. Hence the heading must be changed and the text should be rewritten accordingly.

4) "Data not show" is used several times in the manuscript, however EMBO Molecular Medicine does not permit this (see Author Guidelines: "EMBO Molecular Medicine does not permit citation of "Data not shown". All data referred to in the paper should be displayed in the main or supporting figures. "Unpublished observations" may be referred to in exceptional cases, where these are data peripheral to the major message of the paper and are intended to form part of a future or separate study.")

Additional author correspondence 21 June 2013

We are in the process of finalizing revisions for our manuscript EMM-2013-02655 entitled “An essential role of Decorin in bladder cancer invasiveness” by El Behi et al, for which we received your editorial decision of “major revisions” on April 15th, 2013.

Referee 2 only suggested rewriting in some places, better explanations on the H-Y part of the study, and to avoid the use of ′ data not shown [a]: we will be able to answer all these criticisms, and will provide a set of 5 new supplementary figures (or new panel in a supplementary figure) to document all the former mentions of “data not shown“.

Referee 1 was very concise, and mainly asked for our decorin-related findings to be “repeated in another isogenic set of cell lines, one being aggressive, the other indolent. [...], the mechanism should be documented in at least two independent cell lines”.

To answer this criticism, we have initially searched the literature for other examples of indolent/aggressive bladder tumor cell lines. We did not find any such examples in mice. In human, the group of Theodorescu has described the T24T tumorigenic variants of T24, for which he performed transcriptomic analyses, available in public databases. We have analyzed these data for

DCN expression, and did not observe any significant differences between non-tumorigenic and tumorigenic T24 cells. This model thus does not behave like the MB49/MB49-I model to acquire invasiveness, but this does not invalidate the findings made using our model, and the transcriptomic expression in human bladder tumor biopsies. We did not find any other set of indolent/aggressive human bladder tumor cell lines, all other models corresponding rather to variants of metastatic cell lines with different organ tropisms.
Hence, the option we chose was to create de novo new models of bladder cancer cell lines overexpressing or not DCN, and to analyze their respective invasiveness. Out of 33 human bladder tumor cell lines for which transcriptomic data were available (from our own data set or from publicly available data), we identified 2 with strong expression of the DCN mRNA. We have knocked-down DCN using shRNA in one of these cells, and have demonstrated that this treatment strongly reduces invasive ability of the cells in an in vitro matrigel-invasion assay. We also observed similar behavior of the MB49/MB49-I model in the in vitro matrigel invasion assay (i.e reduced upon DCN knock-down in MB49-I, and increased upon DCN overexpression in MB49).
Despite our efforts, we did not succeed in having the human tumor cell line grow in vivo as xenograft in immunodeficient mice, so we could not provide in vivo demonstration of the pro-invasive activity of DCN, but our in vitro results are very demonstrative. The other human bladder tumor cell line expressing DCN is commercially available from ATCC but currently out of stock until October 2013 (according to ATCC's answer to our enquiry), and its in vivo growth has never been described in the literature, so it would not allow us to rapidly provide in vivo results.

We would now like to ask you for advice on how to proceed further:
if you think that, despite the absence of in vivo new data, our new in vitro results on a human bladder tumor cell line are sufficient to address referee 1's concerns, we will be able to submit our revised article before the 3-months deadline of July 15th.
if you think that in vivo data are mandatory (although such data were not specifically requested in referee 1's comments), we have received a new mouse bladder tumor cell line, which grows slowly in syngeneic mouse hosts (2 months) and which does not express DCN (our own observation). We can try to overexpress DCN in this mouse cell line to see if it improves in vivo growth. But this latter approach, even if successful, will take at least 4 extra months to achieve, hence will make us unable to meet the original deadline for resubmission.

We personally do not think that in vivo data would add more value to our message, especially data obtained in xenografted immunodeficient animals, which would not take into account immune-system dependent mechanisms of tumor progression. We would thus like to submit our revised article with the current set of new data. However, we do not want to risk a negative answer of your referee 1 and a final negative editorial decision from you based on absence of such data.

We thus look forward to hearing your thoughts about this situation, and your preferred option between a resubmission before July 15th with new in vitro data, or a 4 months extension for re-submission with additional (hopefully positive) in vivo data.

Thank you very much for your help in this matter

Additional editorial correspondence 27 June 2013

I now have managed to discuss the issue with the referee.

S/he is not opposed to in vitro data as long as they are properly controlled and are conclusive enough.

As such, I would kindly encourage you to submit your revised manuscript as soon as possible.

1st Revision - authors' response 15 July 2013

Referee #1 (Comments on Novelty/Model System):

The long part of the manuscript on male proteins eliciting an immune response in a female mouse is irrelevant to the clinical setting.

Referee #1 (Remarks):

This paper describes an aggressive variant of an urothelial cancer cell line. The experiments are well selected and carried out in a professional manner.

However, I have reservations regarding two major points:

1. The immune response in females against male surface antigens is only of academic interest and not relevant t a clinical setting. This part of the paper could be reduced dramatically.

We thank this reviewer for his/her positive evaluation of our experimental work, and for his/her comments, which helped us improve our manuscript.

Concerning the comment on novelty and the major point 1 listed in remarks, however, we respectfully disagree with this reviewer, and we apologize for not having expressed in clearer terms in our previous manuscript the relevance of our murine model to the clinical setting.

The male H-Y antigen expressed by the murine bladder tumor cell line MB49 is a prototypical model for neo-antigens resulting from mutations in patient's tumors (e.g point mutations, frameshifts or gene fusions). Since they are not expressed in the healthy host and in thymus during development, such mutated proteins display epitopes that can be recognized by the immune system, like H-Y is recognized by the female immune system.

The H-Y system provides crucial tools to follow specifically an antigen-specific immune response in mice, because of available H-Y-specific $CD4^+$ and $CD8^+$ T cells. These tools allowed us to demonstrate the loss of the model antigen during MB49 progression as the invasive MB49-I variant. But, as highlighted in figure 3D and discussed in the second paragraph of discussion p.15, since the adaptive immune system prevents progression of MB49, but not of MB49-I, also in males, loss of the H-Y antigen alone is not sufficient to explain immune evasion of MB49-I, and this cell line has also most probably lost other unidentified antigens.

Recent data from Next Generation Sequencing show that bladder carcinomas contain high levels of non-silent mutations, and thus of potential neo-antigens: see (Gui et al Nat Genet 2011, 43: 875), and our own analysis of the unpublished TCGA data (http://cancergenome.nih.gov/), showing that 136 bladder tumors contain on average 205 coding mutations/tumor, whereas 3086 human tumors of all origins contain on average 136 coding mutations/tumor.

Therefore, demonstrating a role of immune responses to tumor neo-antigens in bladder cancer is a clinically important issue.

We have thus followed the advice of referee #2 and the editor, stating that "this part would benefit from a much greater explanation and, to our understanding, this would be preferable and indeed improve the manuscript".

In particular, description of immune responses to H-Y in mice bearing MB49 and MB49-I, of the $CD4^+$ (Marilyn) and $CD8^+$ (MataHari) T cell tools, and of the relevance of H-Y as prototypical model for tumor neo-antigens has been included in the results section (p.9). In addition, a sentence has been inserted in the introduction on the neo-antigens in human bladder carcinoma (p.4), and in the second paragraph of discussion p.15 to explain the relevance to human bladder carcinomas of our data on adaptive immune responses to mouse bladder tumors.

2. The findings on Decorin are interesting, but these findings should be repeated in another isogenic set of cell lines one being aggressive, the other indolent. It is not enough to show similar proteins in humans, the mechanism should be documented in at least two independent cell lines.

To answer this criticism, we have initially searched the literature for other examples of indolent/aggressive bladder tumor cell lines. We did not find any such examples in mice. In human, the group of Theodorescu has described the T24T tumorigenic variants of T24 (Gildea et al, Genes Chromosom Cancer, 2000, 27: 252), for which he performed transcriptomic analyses, available in public databases. We have analyzed these data for DCN expression, and did not observe any significant differences between non-tumorigenic and tumorigenic T24 cells. This model thus does not behave like the MB49/MB49-I model to acquire invasiveness, but this does not invalidate the findings made using our model, and the transcriptomic expression in human bladder tumor biopsies. We did not find any other set of indolent/aggressive human bladder tumor cell lines, all other models corresponding rather to variants of metastatic cell lines with different organ tropisms.

Hence, the option we chose was to create de novo new models of bladder cancer cell lines overexpressing or not DCN, and to analyze their respective invasiveness. Out of 33 human bladder tumor cell lines for which transcriptomic data were available (from our own data set, or from publicly available data), we identified 2 with strong expression of the *DCN* mRNA (new figure 7A). One of these cell lines was not available for purchase at ATCC until October 2013, whereas we already had the other one (TCCSUP) in our laboratory. We have knocked-down DCN using shRNA in TCCSUP (new figure 7B). In our hands, TCCSUP cells did not form tumors when injected subcutaneously in immunodeficient mice (we tried up to 10^7 cells, without or with matrigel, in either Nude or SCID mice), so we could not investigate the role DCN *in vivo.* However, the study of genes correlated with DCN in muscle-invasive bladder carcinoma suggested a pro-invasive effect of decorin, which we therefore investigated *in vitro*. We now demonstrate that silencing decorin strongly reduces invasive ability of the cells in an *in vitro* matrigel-invasion assay (new figure 7C-D). We also observed similar behavior of the MB49/MB49-I model in the *in vitro* matrigel invasion assay (i.e reduced upon DCN knock-down in MB49-I, and increased upon DCN overexpression in MB49, new supplementary Figure S8).

Thus, the new results shown in figures 7 and supplementary S8 (p.14-15, discussion p.19) provide the requested documentation of the mechanism described here in 2 different cell lines, including a human bladder tumor cell, and we hope that this reviewer will positively consider this new addition to our work.

The abstract has been modified to describe these new results.

Referee #2 (Comments on Novelty/Model System):

The functional data that prove the key finding are generated in a murine system with an artificially implanted cell-line-derived tumor. The data are backed up by a descriptive analysis with corresponding human material, but as the key finding is very novel is can not yet be said, whether the murine system is suitable

Referee #2 (Remarks):

The manuscript by Mohamed El Behi et al. examines why the aggressive bladder carcinoma line MB49-I and its less aggressive parental line are different in their invasiveness. They pin it down to the expression of certain antigens (esp. H-Y) but, more importantly, to the expression of the

proteoglycan decorin by the more aggressive variant. This is the key finding of this paper.

The key finding that decorin is sufficient and necessary for the observed enhanced tumor growth in vivo is new and interesting, because decorin-expression so far has been associated with reduced malignancy. The experiments performed to show this are well designed; appropriate controls and statistical analysis were performed. Although an artificial murine model system was used, the relevance for human bladder carcinoma is indicated by a correlative analysis of human patient material.

Unfortunately, the way parts of the manuscript (esp. at the beginning) are written does not comply with the good experimental work of the authors. Some experiments are difficult to understand for anyone, who did not perform them, and the introduction contains information completely irrelevant to understand the paper, but lacks other necessary explanations.

Hence I conclude that a revised manuscript would be well worth publishing in EMBO Molecular Medicine, and that no additional experiments are needed, but that some parts of the manuscript must be rewritten to be more accurate and to make the work better understandable for the general readership.

We thank this reviewer for all these very positive comments, and his/her specific remarks and suggestions, which we have followed to improve the presentation and interpretations of our results.

As detailed below, we have extensively re-written our manuscript to answer points 1-3. We have also included 5 supplementary figures and one supplementary panel to actually show the data mentioned as “data not shown” in the previous manuscript (point 4).

In addition, to answer the other reviewer’s criticism, a new figure showing the effect of decorin in invasiveness of a human bladder tumor cell line has been included in the revised manuscript (new Fig.7 and new suppl fig.8). The abstract has been slightly modified to describe these new data.

Precise points:

1) The introduction does not say anything about decorin, although it is the key molecule of the manuscript. The function and role of decorin must be covered in the introduction.

We have now inserted in the introduction of the revised manuscript a paragraph describing the known functions of decorin in cancer (p.5).

BCG has nothing to do with the described experiments, so it should not be covered in that detail.

We agree with this reviewer that description of the immunological effects of BCG in bladder cancer treatments is not necessary to introduce our work, and we have thus deleted three sentences in the first and second paragraph of introduction (p.4), to shorten this part.

The last paragraph of the introduction is in principle another abstract and hence redundant. If space is required, it can be omitted.

Since we have now expanded the introduction to describe decorin, we have followed this reviewer’s advice and considerably shortened the last paragraph of introduction, which now very succinctly presents the major points of our article, rather than describing them in too much details.

2) The explanation of figure 2 in the result section (page 7) in insufficient. What does the first panel (CD45.2 of CD45) tell? Did the authors stain for CD4/FoxP3 double positive or just for FoxP3 (it is differently described in the text and the figure).

We thank this reviewer for his/her detailed analysis of figure 2, and indeed, we apologize for having mislabelled some of the panels, making the figure difficult to understand.

In Figure 2A, the label "CD45.2$^+$ cells" has now been changed to "total immune cells": this panel shows the percentage of CD45$^+$ = hematopoietic cells infiltrating either MB49 or MB49-I tumors (described as "overall percentage of total immune cells" in the 2nd paragraph of results section). The label "foxp3$^+$ cells" has been corrected to "Treg": this panel shows the percentage of regulatory CD4$^+$ Foxp3$^+$ cells (of CD45$^+$ cells), as now indicated as legend of the vertical axis and in the results section (regulatory CD4$^+$/Foxp3$^+$ cells).

The panel "CD19$^+$ cells" has been corrected to "B cells", and CD8$^+$/Foxp3$^+$ T cells have been corrected to "CD8$^+$/Treg".

We hope that this figure is now clear to the reader

Since the MB49-tumors are already regressing while the MB49-I ones are growing, the observed differences may simply show the infiltration into a regressing vs. a growing tumor. The whole paragraph should be rewritten. The figure legend of figure 2 does not explain the figure properly, and should be more detailed.

We agree that the difference in immune infiltration at d10-12 between MB49 and MB49-I may simply reflect the difference between a regressing (MB49) versus a growing (MB49-I) tumor. But since regression does not take place in mice devoid of T and B cells (Figure 1, MB49 growth in Rag$^{-/-}$ mice), the observed specific increase of CD8+ T cells as compared to Treg in MB49 suggests that an efficient cytotoxic T cell response occurs in MB49.

A sentence explaining this interpretation has been inserted at the end of the paragraph, describing figure 2 (p.8-9), and the beginning of this paragraph now clearly explains that no difference in immune infiltration is observed at d5 post-tumor implantation, when both tumors are equally growing (p.8). In addition, legend of Figure 2 has been expanded to explain in more details the experimental setting and the nature of the immune cells analyzed.

3) On page 8, result section, it is implied (even though it is not explicitly stated) that H-Y was lost due to immune surveillance pressure during successive passages, however, as described later, this was done in male mice. The tumors ability to activate CD4 and CD8 T cells was examined with the Marilyn and MataHari system, which only detect H-Y-antigens (and should be briefly explained here). These were lost independently of immunological pressure, so the authors cannot conclude that the tumor lost its general ability to stimulate T cells. They can only conclude, from the differences observed in the RAG KO mice that the adaptive immune system does, or does not play a role. Hence the heading must be changed and the text should be rewritten accordingly.

We apologize for an apparently misleading text in the results section p.8 of the previous manuscript.

On p.9 of the revised manuscript, we have changed the heading of this paragraph to specify that MB49-I has lost ability to activate H-Y-specific (rather than more generally antigen-specific) T cells. We have also included 2 sentences to describe the use of H-Y as a model for neo-antigens arising from mutations in tumors, and to describe the Marilyn (CD4$^+$) and MataHari (CD8$^+$) H-Y-specific T cells in the results (in addition to their description in the materials and methods).

We did not want to imply that selective immune pressure was the cause of H-Y loss in this tumor, and we have now deleted "during successive in vivo passages" from the last sentence of the 1st paragraph of this result section to avoid misleadingly suggesting it. We have also now clearly

explained in the introduction, p.6 first paragraph, that MB49 is of male origin and was passaged in male hosts to give rise to MB49-I.

Finally, the last set of data presented in this paragraph show that the adaptive immune system limits MB49 growth independently of H-Y antigens, which suggest either the loss of other tumor antigens during passages in vivo (as proposed in the previous manuscript), or loss of the general capacity to stimulate adaptive immune cells (as suggested by the reviewer). We have changed the conclusion sentence of this paragraph p.10 to include this last hypothesis, as suggested by the reviewer.

4) "Data not show" is used several times in the manuscript, however EMBO Molecular Medicine does not permit this (see Author Guidelines: "EMBO Molecular Medicine does not permit citation of "Data not shown". All data referred to in the paper should be displayed in the main or supporting figures. "Unpublished observations" may be referred to in exceptional cases, where these are data peripheral to the major message of the paper and are intended to form part of a future or separate study.")

We apologize for our previous too extensive use of "data not shown".

In the revised manuscript, all the previous "data not shown" are now illustrated by additional supplementary figures (1 new panel in former suppl Fig S3 now suppl Fig S5, plus 5 new supplementary figures = 9 suppl figures in total).

2nd Editorial Decision 09 August 2013

Thank you for the submission of your revised manuscript to EMBO Molecular Medicine. We have now received the enclosed reports from the referees that were asked to re-assess it. As you will see the reviewers are now globally supportive and I am pleased to inform you that we will be able to accept your manuscript pending the following final amendments:

- As per our Author Guidelines, the description of all reported data that includes statistical testing must state the name of the statistical test used to generate error bars and P values, the number (n) of independent experiments underlying each data point (not replicate measures of one sample), and the actual P value for each test (not merely 'significant' or 'P < 0.05').

- Please provide a final version of your manuscript without the coloured lettering (no longer needed)

- Please remove the supplementary information figure legends from the main text

Please submit your revised manuscript within two weeks. We look forward to seeing a revised form of your manuscript as soon as possible.

***** Reviewer's comments *****

Reviewer #1

Is suitable for publication

The functional data that prove the key finding are generated in an murine system with an artificially implanted cell-line-derived tumor. The data are backed up by a descriptive analysis with corresponding human material, but as the key finding is very novel is can not yet be said, whether the murine system is suitable

Reviewer #2
Essential corrections have been made so I am willing to accept

Additional Editorial Correspondence 14 August 2013

Thank you very much for resubmitting your manuscript and indicating when appropriate p values and which statistical test was used in legends.

I also noticed that you have not indicated the microarray accession number of the experiments performed in this study. Please submit your data to GEO or ArrayExpress if you already have not done so and add the accession number within the materials and methods section prior to uploading the final text.

We will put back the article into the "author approval" folder so you will be able to easily reload the modified final file(s).

Revision received and accepted 06 September 2013

© EMBO
