# Supplementary material for: An essential role for decorin in bladder cancer invasiveness
Source: EMBO Mol Med. 2013 Oct 20;5(12):1835–51. doi: 10.1002/emmm.201302655 (PMC3914526; doi:10.1002/emmm.201302655)
Supplement: Supplementary file 2 [file emmm0005-1835-sd2.pdf]

Supplementary materials for

An essential role for decorin in bladder cancer invasiveness

M. El Behi, S. Krumeich, C. Lodillinsky, A. Kamoun, L. Tibaldi, G. Sugano, A. De Reynies, E. Chapeaublanc, Y. Allory, F. Radvanyi, O. Lantz, A. M. Eiján, I. Bernard-Pierrot*, C. Théry*

Table of content

Supplementary figure 1: Analysis of immune cells infiltrating bladder five days after tumor injection

Supplementary figure 2: necrotic MB49-I cells do not activate HY-specific T cells

Supplementary figure 3: MB49 grows faster than MB49-I *in vitro*

Supplementary figure 4: Decorin knockdown in MB49-I cells does not affect the nature of immune cells infiltrating the tumor

Supplementary figure 5: Two additional independent analyses confirm that decorin is overexpressed in human MIBC as compared to NMIBC.

Supplementary figure 6: *DCN* mRNA level is not correlated to DCN DNA level in bladder tumors.

Supplementary figure 7: Gene ontology annotation enriched within genes correlated to *DCN.*

Supplementary figure 8: Decorin is not required for *in vitro* proliferation of TCCSUP, and promotes matrigel invasion by MB49 and MB49-I cells *in vitro*.

Supplementary figure 9: a specific DCN isoform is expressed by muscle cells in bladder.

Supplementary Table 1: Genes with mRNA expression level correlated or anticorrelated to *DCN* expression level in human MIBC.

Supplementary Table 2: Functional pathways and gene ontology annotations relative to biological processes significantly enriched in genes positively correlated with *DCN* in human MIBC.

Supplementary Table 3: Affymetrix U133plus2.0 microarray data in 162 human bladder tumors and 4 normal samples, for genes correlated with *DCN* in MIBC (pearson R>0.5).

Supplementary figure 1: Analysis of immune cells infiltrating bladder five days after tumor injection

Nature of immune lymphoid cells infiltrating MB49 and MB49-I 5 days after orthotopic injection of the tumors, analyzed by multicolour flow cytometry as described in figure 2. Percent of each cell type in individual mice is indicated (pool of 2 independent experiments). No difference is observed in the immune infiltrates of the two tumors at this time point.

Supplementary Figure 1

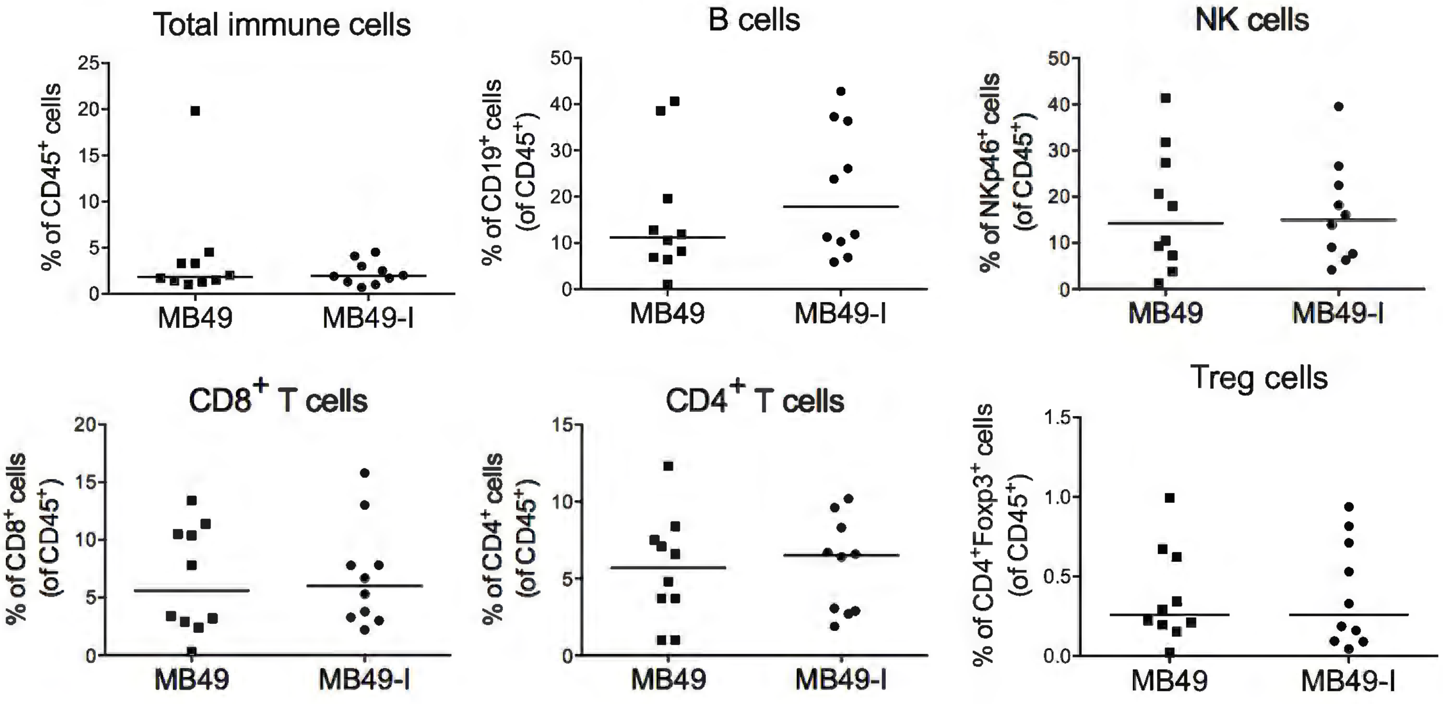

Supplementary figure 2: necrotic MB49-I cells do not activate HY-specific T cells

A: After repeated cycles of freezing and thawing, MB49 and MB49-I cells emulsified in CFA were injected in the footpads of CD45.1 female B6 mice. Mice were injected 24h later with 4.10^6 CellTrace Violet-labeled CD45.2 splenocytes from Marilyn and MataHari mice. Six days after the initial injection of protein extracts, mice were sacrificed and CellTrace Violet dilution was analyzed in $CD45.2^+$ $V\beta6^+CD4^+$ (Marilyn) or $V\beta8^+CD8^+$ (MataHari) T cells from popliteal lymph nodes. B: Quantification of the % of dividing Marilyn and MataHari cells from one experiment with two mice in each group.

Supplementary Figure 2

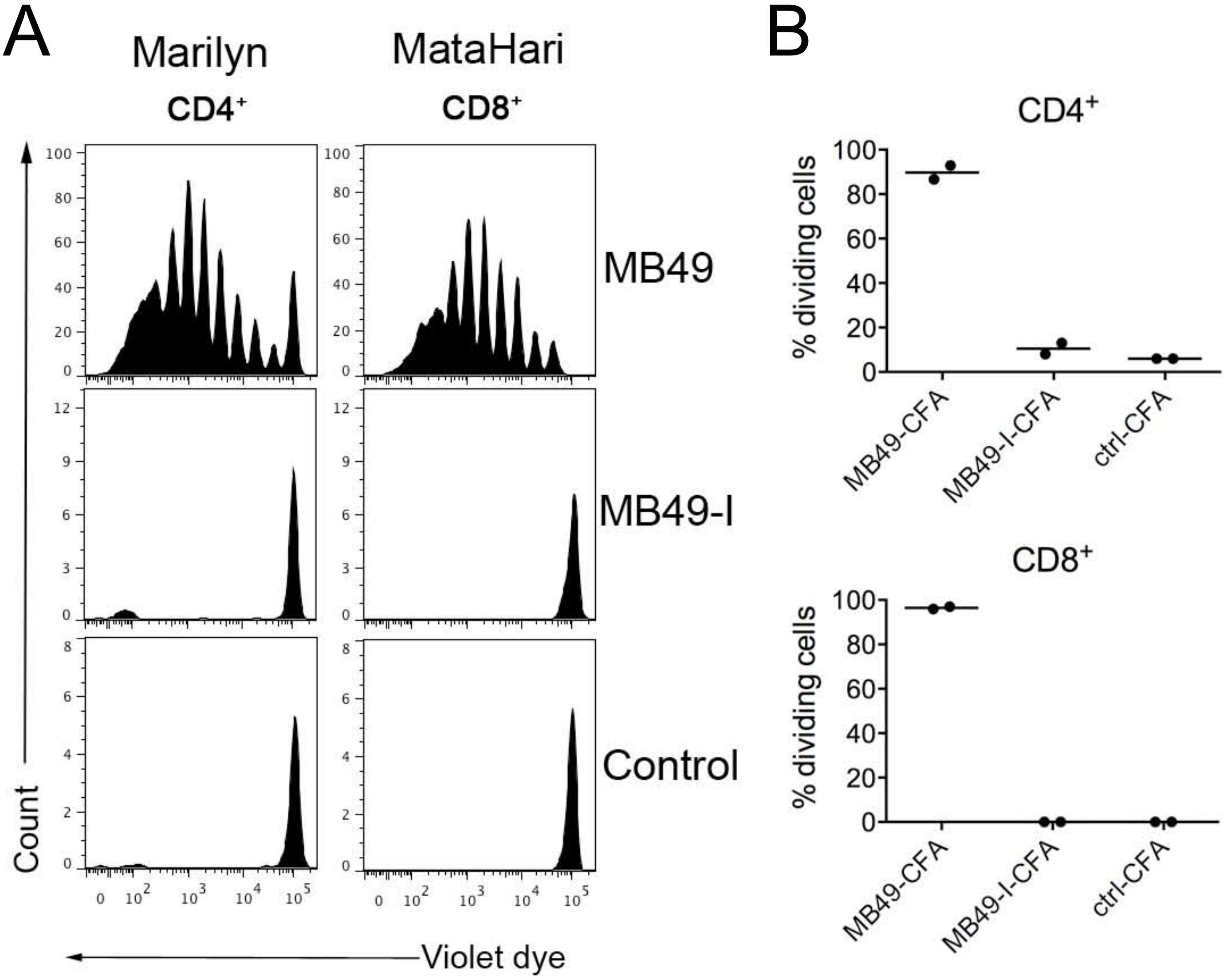

Supplementary figure 3: MB49 grows faster than MB49-I *in vitro*

In vitro growth of MB49 and MB49-I cells quantified during 48h using Cell Titer blue viability assay (pool of 2 independent experiments). **: p= 0.0042 at 24h, p=0.0076 at 48h (Mann-Whitney test).

Supplementary Figure 3

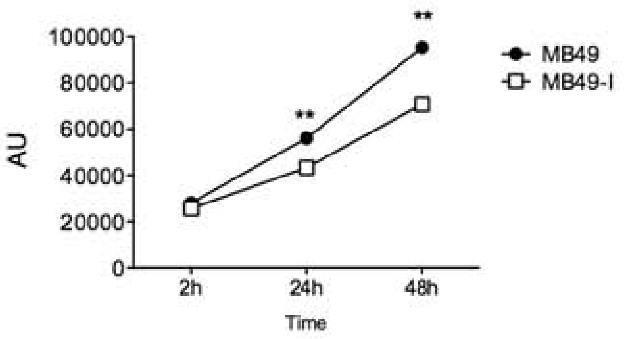

Supplementary figure 4: Decorin knockdown in MB49-I cells does not affect the nature of immune cells infiltrating the tumor

Nature of immune lymphoid cells infiltrating MB49-I-shDcn1, MB49-I-shDcn2 and MB49-I-Scr after orthotopic injection of the tumors, analysed by multicolour flow cytometry as in Figure 2 and Supplementary Figure 1. Seven days after injection, hematopoietic cells infiltrating the bladder were defined by their expression of the pan-hematopoietic marker CD45. Percent of each hematopoietic cell type in individual mice is indicated. Data from one experiment with six mice per group is shown. Horizontal bar = median. n.s. = non significant as compared to Scr (total cells: p>0.9999 for shDcn1 and shDcn2; B cells: p>0.9999 for shDcn1, p=0.4469 for shDcn2; NK cells: p=0.25 for shDcn1, p>0.9999 for shDcn2; $CD4^+$ T cells: p=0.1301 for shDcn1, p>0.9999 for shDcn2; $CD8^+$ T cells: p=0.25 for shDcn1, p>0.9999 for shDcn2; Treg cells: p=0.25 for shDcn1 and shDcn2) (Mann-Whitney test).

Supplementary Figure 4

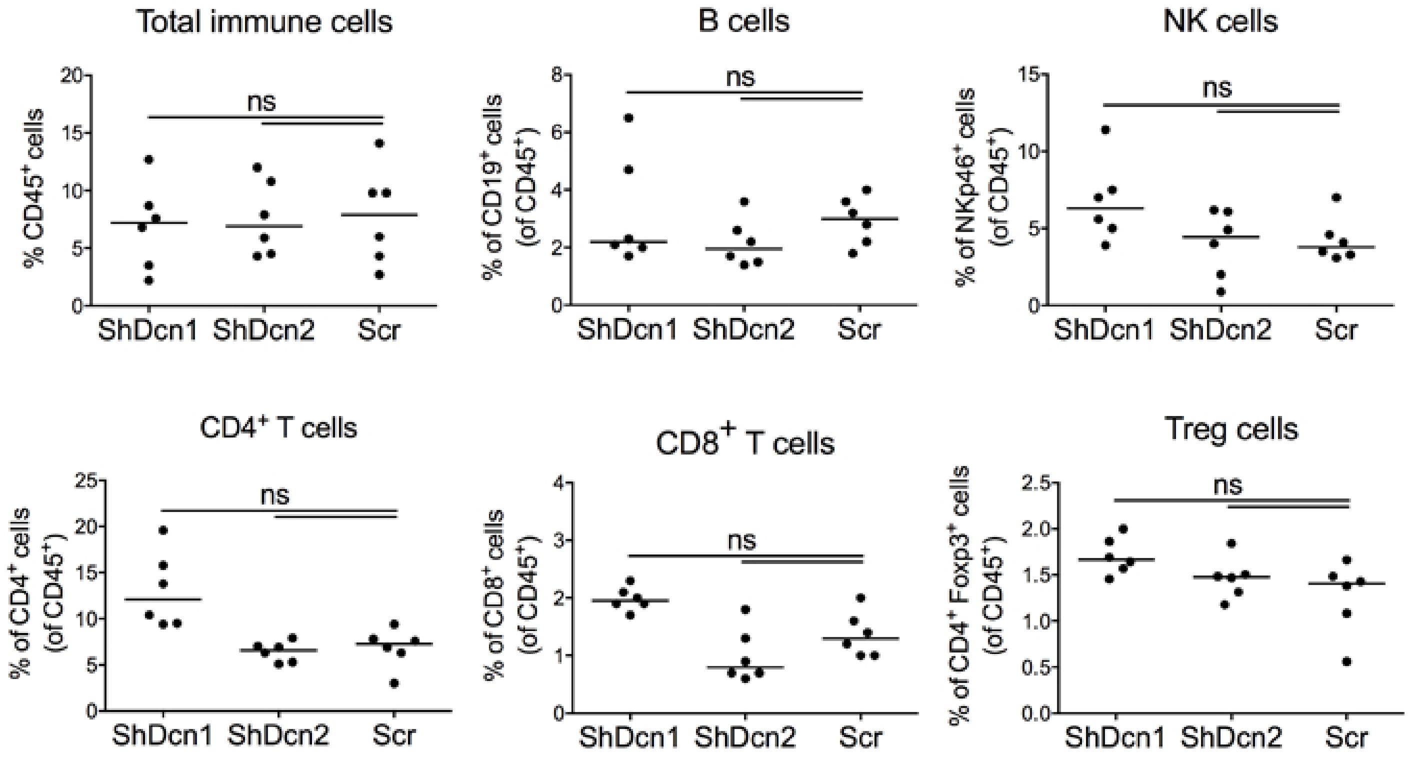

Supplementary figure 5: Two additional independent analyses confirm that decorin is overexpressed in human MIBC as compared to NMIBC.

Decorin **(***DCN)* mRNA expression was determined A: in our CIT set of 176 human bladder tumors using Affymetrix exon1.0st array and B: in three independent publicly-available datasets of human bladder tumors (Sanchez-Carbayo et al, 2006; Lindgren et al, 2010; Riester et al, 2012). Ta, T1 = non-muscle-invasive bladder carcinoma (NMIBC); T234 = Muscle-Invasive bladder carcinoma (MIBC).

Supplementary Figure 5

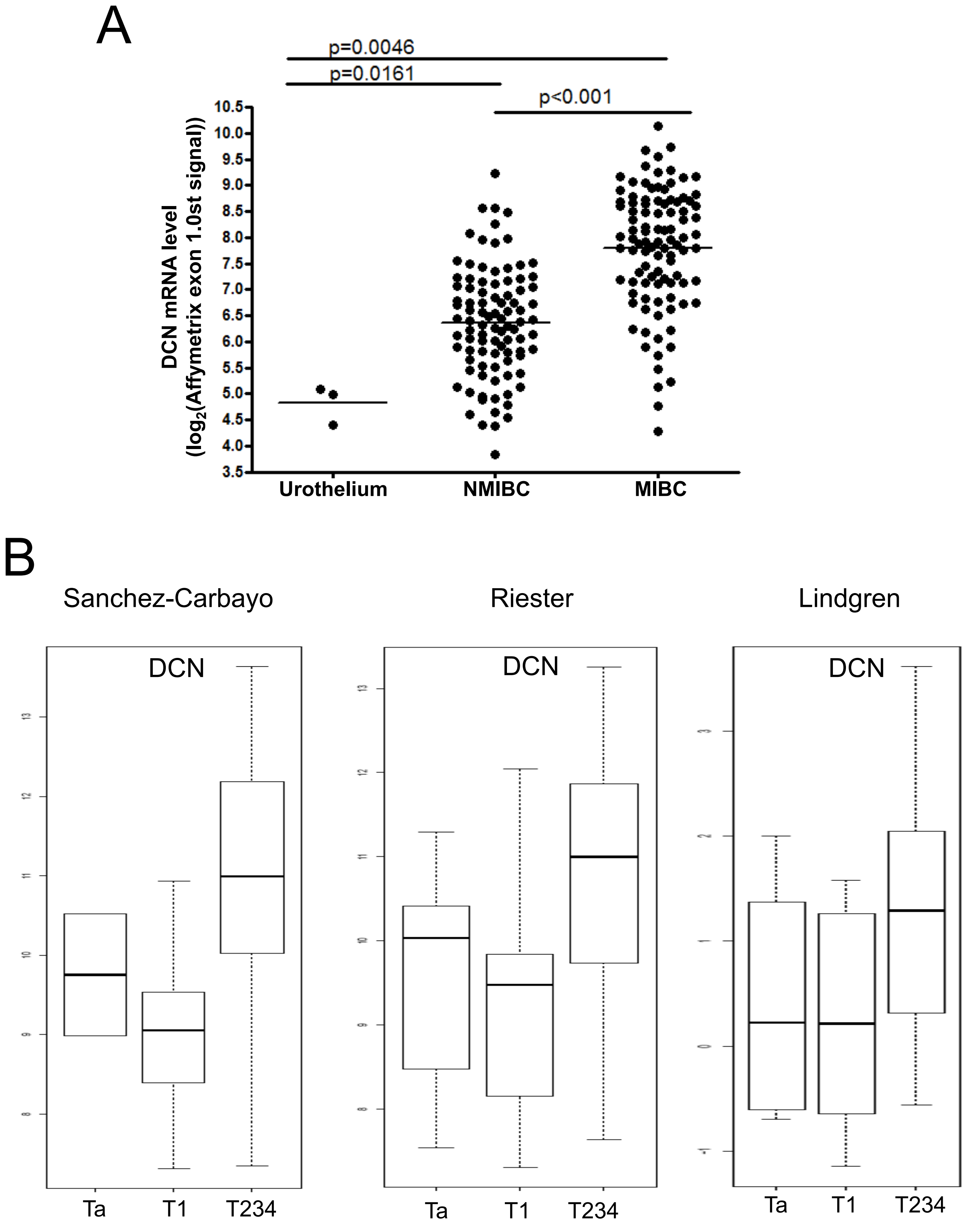

Supplementary figure 6: *DCN* mRNA level is not correlated to DCN DNA level in bladder tumors.

Linear regression analysis of the 153 bladder carcinomas for which we had both comparative genomic hybridization (CGH) and expression data. The Affymetrix signal (U133plus2.0) and the CGH log_2-ratio (Log_2(DNA copy number/2)) were compared, using Spearman's correlation test. Expression levels were considered significantly modified with respect to DNA copy number if $p < 0.05$.

Supplementary Figure 6

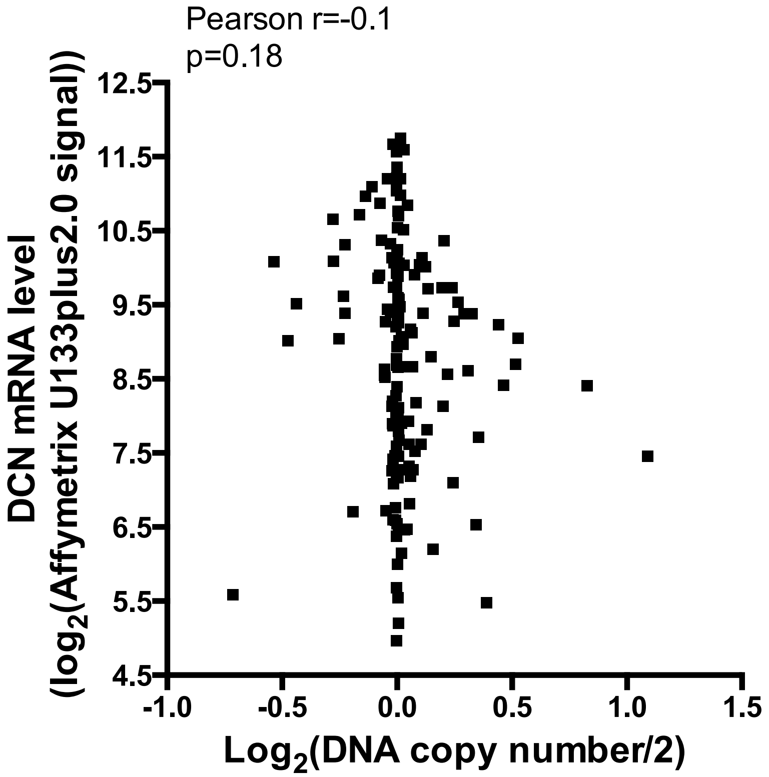

Supplementary figure 7: Gene ontology annotation enriched within genes correlated to *DCN*.

GO enrichment in genes positively correlated to *DCN* in human MIBC (pearson's correlation, $p<1.10^{-6}$ and $R>0.5$) was determined using GoRilla application ($p\text{-value}<1.10^{-9}$).

Supplementary figure 7

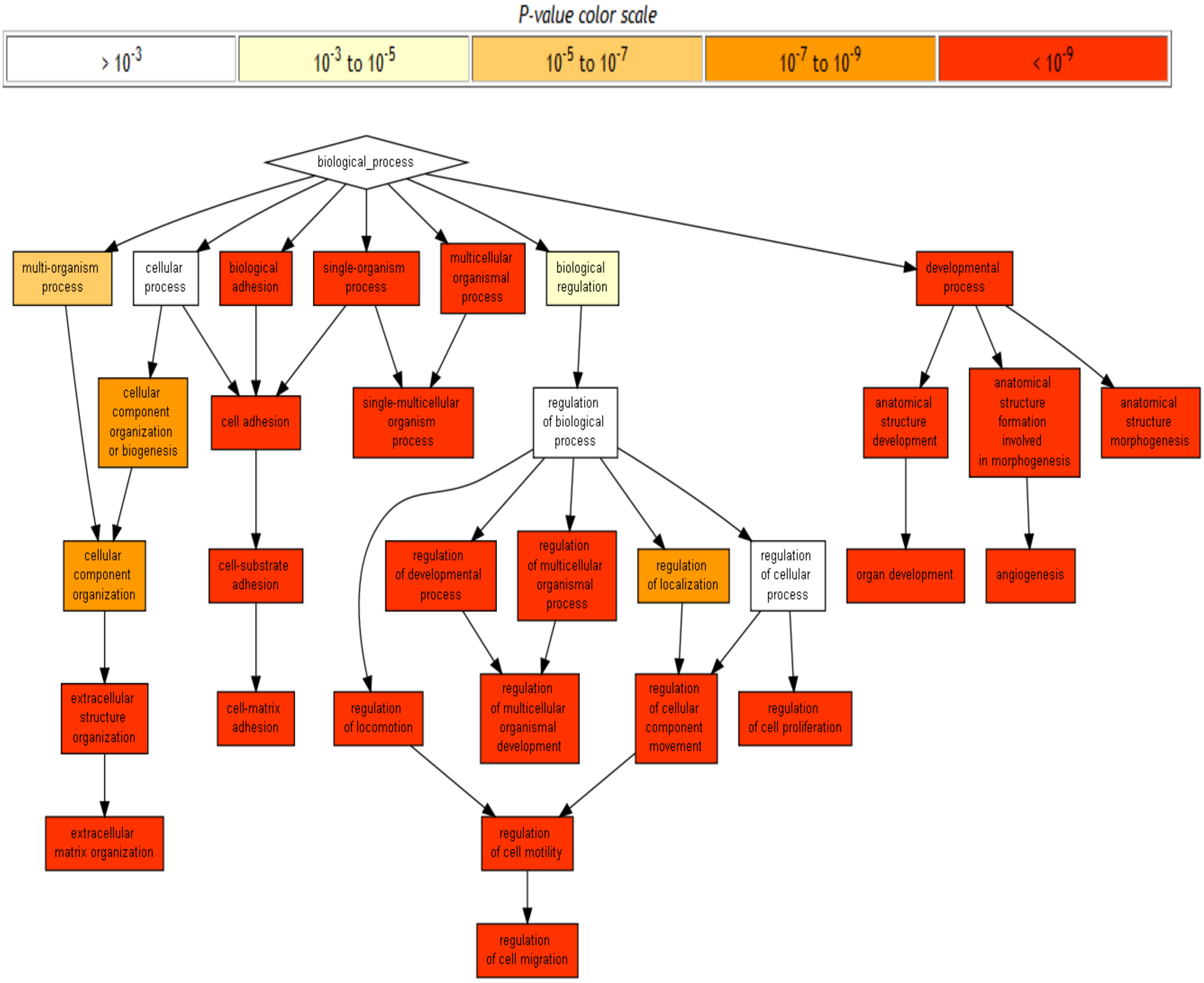

Supplementary figure 8: Decorin is not required for *in vitro* proliferation of TCCSUP, and promotes matrigel invasion by MB49 and MB49-I cells *in vitro*.

A: *In vitro* growth of TCCSUP transduced with shDCN1, shDCN2 or Scr quantified during 48h using Cell Titer blue viability assay.

B-C: Quantification of matrigel invasion by MB49 and MB49-I using the xCELLigence device. Cells were seeded in the upper chamber containing matrigel of CIM-plates. Slope values of the cell index was calculated 36h after cell seeding, in 2-3 individual wells of 4 independent experiments. B: quantification of slope values in MB49-Dcn as compared to MB49-pCMV (considered as 100%). *: p=0.031, 2-way ANOVA followed by Tukey's post-Hoc test (on raw values of each experiment, with medium alone as additional condition). C: Quantification of slope values in MB49I-shDcn1 and MB49-I-shDcn2 as compared to MB49-I-Scr (considered as 100%). *: p=0.024 for shDcn1 compared to Scr, p=0.06 for shDcn2 compared to Scr, 2-way ANOVA followed by Tukey's post-Hoc test (on raw values of each experiment, with medium alone as additional condition).

Supplementary Figure 8

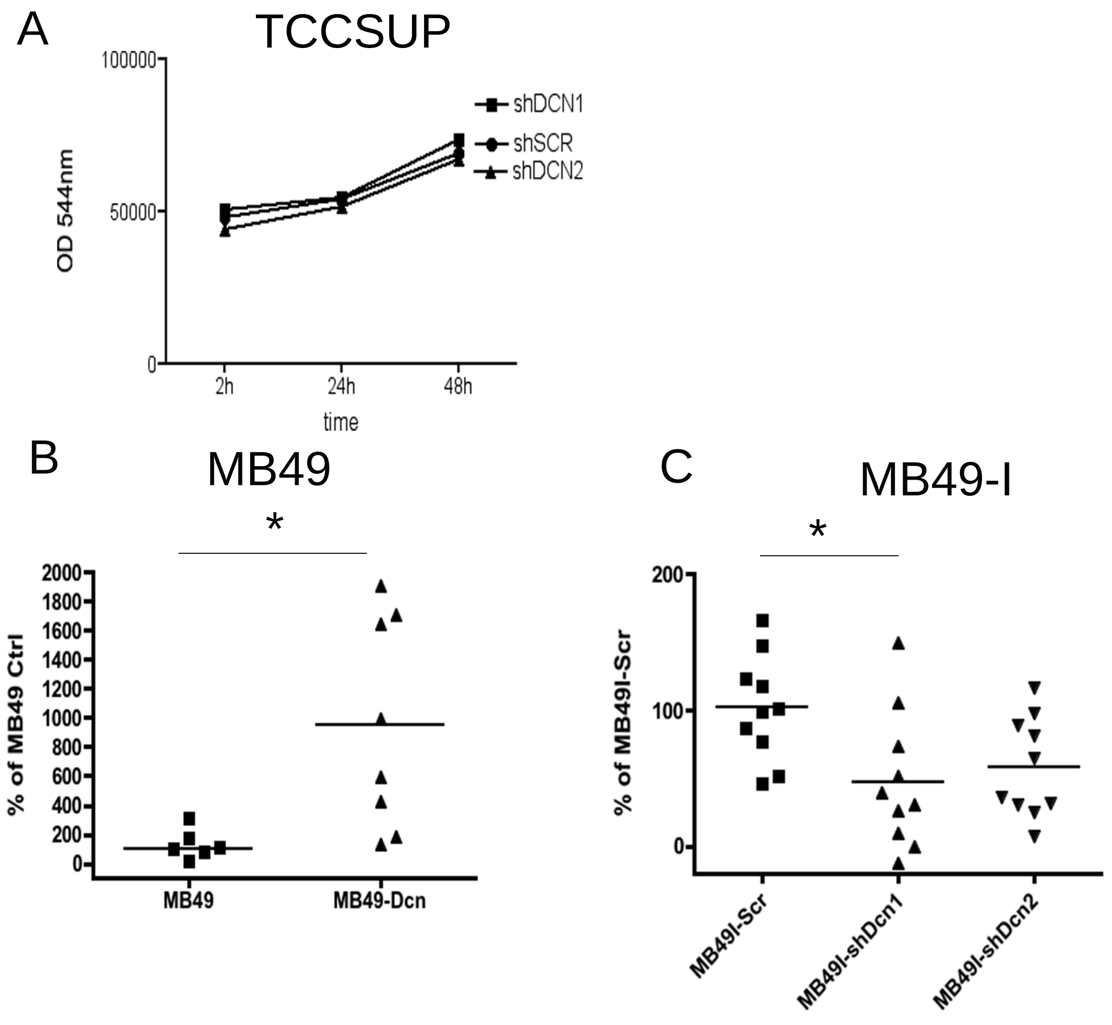

Supplementary figure 9: a specific DCN isoform is expressed by muscle cells in bladder.

A: Decorin (*DCN*) mRNA expression was measured by Affymetrix U133plus2.0 array in urothelium and muscle from bladder samples. B: Expression of decorin (DCN) protein in a section of a normal bladder showing absence of labeling in urothelial cells but strong expression by smooth muscle cells. Scale bar 100 µm. Picture was extracted from the human protein atlas portal (http://www.proteinatlas.org/). C: DCN exon array profiles from 106 MIBC tumors (red), 101 NMIBC tumors (green), 4 normal urothelium samples (blue) and 5 bladder muscle samples (purple). Individual sample expression values are plotted for each targeted exon on Affymetrix exon1.0st array, and drawn lines join the median values for each sample group on each exon. Transcripts 1 and 2 are known transcripts referenced in Ensembl database (http://www.ensembl.org/). The third transcript structure suggests the use in urothelial cells of several putative first exons already referenced by Ensembl. First exons of transcripts 1 and 2 seem to be only expressed in muscle samples. We therefore hypothesize that urothelial samples rather express transcripts using one of the first exons drawn in black. The observed overexpression of DCN gene in MIBC samples does not correspond to an overexpression of transcript 1 and 2 given their first exons expression profile. This ensures that overexpression of DCN in MIBC is not the result of a contamination by muscle cells.

Supplementary Figure 9

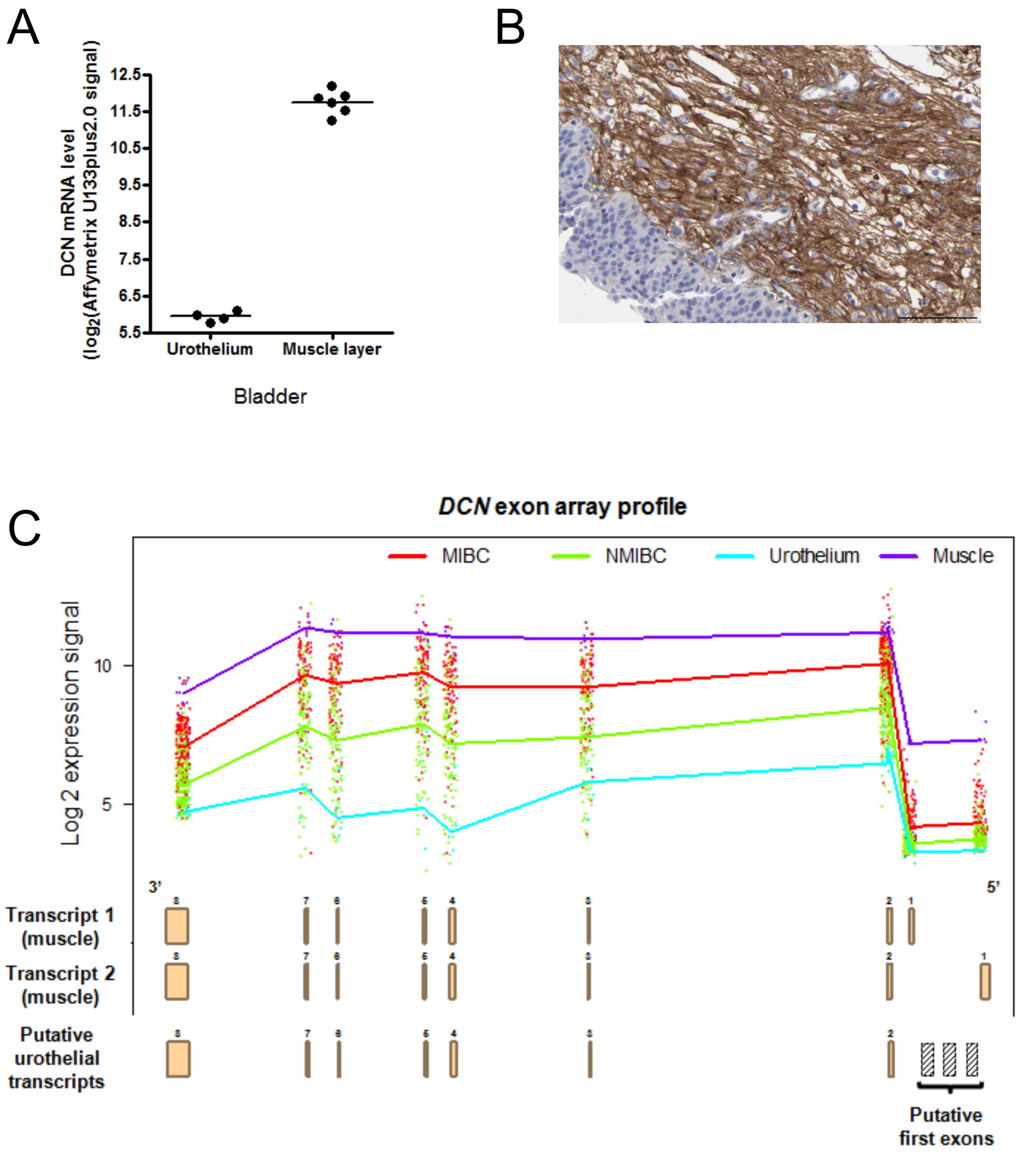

Supplementary Table 1: Genes with mRNA expression level correlated or anticorrelated to *DCN* expression level in human MIBC.

Threshold p-value<1.10^{-6}, [R] > 0.5.

Supplementary Table1:
Genes with mRNA expression level correlated or anticorrelated to DCN expression level in human MIBC.
Threshold p<1E-6, |R|>0.5

Symbol	Gene name	Pearson	P-value
DCN	decorin	1	0
LUM	lumican	0,910764984	0
CDH11	cadherin 11, type 2, OB-cadherin (osteoblast)	0,90145749	0
COL6A3	collagen, type VI, alpha 3	0,899155107	0
SERPINF1	serpin peptidase inhibitor, clade F (alpha-2 antiplasmin, pigment epithelium derived factor), member 1	0,871870156	0
RARRES2	retinoic acid receptor responder (tazarotene induced) 2	0,863819733	0
COL3A1	collagen, type III, alpha 1	0,860517682	0
ACTA2	actin, alpha 2, smooth muscle, aorta	0,857753487	0
SPARC	secreted protein, acidic, cysteine-rich (osteonectin)	0,856986043	0
FBN1	fibrillin 1	0,849373187	0
HEPH	hephaestin	0,846801428	0
COL1A2	collagen, type I, alpha 2	0,842476651	0
CTSK	cathepsin K	0,841029438	0
GLT8D2	glycosyltransferase 8 domain containing 2	0,836292453	0
TAGLN	transgelin	0,835657024	0
COL6A2	collagen, type VI, alpha 2	0,834324908	0
SPON1	spondin 1, extracellular matrix protein	0,832498364	0
SFRP2	secreted frizzled-related protein 2	0,829617806	0
MMP2	matrix metallopeptidase 2 (gelatinase A, 72kDa gelatinase, 72kDa type IV collagenase)	0,829464196	0
CD248	CD248 molecule, endosialin	0,821211379	0
COL5A1	collagen, type V, alpha 1	0,819333126	0
GPR124	G protein-coupled receptor 124	0,817253785	0
MYLK	myosin light chain kinase	0,816676378	0
PCOLCE	procollagen C-endopeptidase enhancer	0,814330236	0
OLFML1	olfactomedin-like 1	0,812065175	0
CALD1	caldesmon 1	0,810365928	0
PALLD	palladin, cytoskeletal associated protein	0,808822783	0
PDLIM3	PDZ and LIM domain 3	0,804765357	0
DDR2	discoidin domain receptor tyrosine kinase 2	0,803772521	0
FILIP1L	filamin A interacting protein 1-like	0,802232185	0
OLFML3	olfactomedin-like 3	0,799776397	0
AEBP1	AE binding protein 1	0,796061551	0
CCDC80	coiled-coil domain containing 80	0,793936246	0
POSTN	periostin, osteoblast specific factor	0,791791302	0
LHFP	lipoma HMGIC fusion partner	0,790407917	0
C1S	complement component 1, s subcomponent	0,787778931	0
COL1A1	collagen, type I, alpha 1	0,787460844	0
MYL9	myosin, light chain 9, regulatory	0,784395387	0
CRISPLD2	cysteine-rich secretory protein LCCL domain containing 2	0,781132715	0
ANGPTL2	angiopoietin-like 2	0,780900441	0
PDGFRB	platelet-derived growth factor receptor, beta polypeptide	0,777708178	0
COL15A1	collagen, type XV, alpha 1	0,775416911	0
HTRA1	HtrA serine peptidase 1	0,775202897	0
ACTG2	actin, gamma 2, smooth muscle, enteric	0,774548563	0
EMILIN1	elastin microfibril interfacer 1	0,772520548	0
MXRA8	matrix-remodelling associated 8	0,771987478	0
ECM2	extracellular matrix protein 2, female organ and adipocyte specific	0,770057236	0
LRRC32	leucine rich repeat containing 32	0,770008715	0
TGFB3	transforming growth factor, beta 3	0,76990014	0
TCF4	transcription factor 4	0,766397398	0
EFEMP2	EGF-containing fibulin-like extracellular matrix protein 2	0,766240054	0
NID2	nidogen 2 (osteonidogen)	0,76458498	0
TGFB1I1	transforming growth factor beta 1 induced transcript 1	0,760609978	0
ZNF521	zinc finger protein 521	0,759753366	0
GREM1	gremlin 1, cysteine knot superfamily, homolog (Xenopus laevis)	0,758842524	0
VCAN	versican	0,753934739	0
LAMA2	laminin, alpha 2	0,753773336	0
COL6A1	collagen, type VI, alpha 1	0,753451175	0

C1R	complement component 1, r subcomponent	0,751236769	0
PXDN	peroxidasin homolog (Drosophila)	0,751212158	0
CXCL12	chemokine (C-X-C motif) ligand 12 (stromal cell-derived factor 1)	0,751170054	0
EDNRA	endothelin receptor type A	0,750066165	0
COL16A1	collagen, type XVI, alpha 1	0,7499073	0
PDGFRA	platelet-derived growth factor receptor, alpha polypeptide	0,748544459	0
LDB2	LIM domain binding 2	0,747017214	0
SPARCL1	SPARC-like 1 (hevin)	0,74609417	0
TIMP2	TIMP metallopeptidase inhibitor 2	0,744101397	0
THBS2	thrombospondin 2	0,743365479	0
SMOC2	SPARC related modular calcium binding 2	0,743157324	0
MSRB3	methionine sulfoxide reductase B3	0,74306473	0
AOC3	amine oxidase, copper containing 3 (vascular adhesion protein 1)	0,741318866	0
ANTXR1	anthrax toxin receptor 1	0,736314716	0
COL5A2	collagen, type V, alpha 2	0,736252012	0
COX7A1	cytochrome c oxidase subunit VIIa polypeptide 1 (muscle)	0,736051819	0
CNRIP1	cannabinoid receptor interacting protein 1	0,735952487	0
THY1	Thy-1 cell surface antigen	0,735508242	0
VIM	vimentin	0,735414534	0
ANXA6	annexin A6	0,735138358	0
IGFBP7	insulin-like growth factor binding protein 7	0,734560609	2,22E-16
PRRX1	paired related homeobox 1	0,733576137	2,22E-16
EBF1	early B-cell factor 1	0,732159546	2,22E-16
MEG3	maternally expressed 3 (non-protein coding)	0,731226351	2,22E-16
FMOD	fibromodulin	0,73087928	4,44E-16
ZCCHC24	zinc finger, CCHC domain containing 24	0,729722695	4,44E-16
COL14A1	collagen, type XIV, alpha 1	0,727613073	4,44E-16
PRICKLE2	prickle homolog 2 (Drosophila)	0,726122011	4,44E-16
LOC387763	hypothetical protein LOC387763	0,723820033	8,88E-16
C4orf18	chromosome 4 open reading frame 18	0,72267347	8,88E-16
MXRA5	matrix-remodelling associated 5	0,722047478	8,88E-16
LMCD1	LIM and cysteine-rich domains 1	0,721000611	1,11E-15
IGDCC4	immunoglobulin superfamily, DCC subclass, member 4	0,720546115	1,33E-15
C1QTNF5	C1q and tumor necrosis factor related protein 5	0,719697295	1,33E-15
RHOJ	ras homolog gene family, member J	0,716822955	1,78E-15
KCTD12	potassium channel tetramerisation domain containing 12	0,716099548	2,22E-15
MFAP4	microfibrillar-associated protein 4	0,713428455	3,11E-15
TPM2	tropomyosin 2 (beta)	0,711900441	3,55E-15
PTRF	polymerase I and transcript release factor	0,711444548	4,00E-15
FBLN5	fibulin 5	0,710908386	4,22E-15
ELTD1	EGF, latrophilin and seven transmembrane domain containing 1	0,710787132	4,22E-15
ABI3BP	ABI family, member 3 (NESH) binding protein	0,710168968	4,44E-15
MRVI1	murine retrovirus integration site 1 homolog	0,709217774	5,33E-15
MYADM	myeloid-associated differentiation marker	0,707357686	6,66E-15
MRC2	mannose receptor, C type 2	0,706943212	6,88E-15
ZFPM2	zinc finger protein, multitype 2	0,705834017	7,99E-15
VCAM1	vascular cell adhesion molecule 1	0,702712741	1,15E-14
ISLR	immunoglobulin superfamily containing leucine-rich repeat	0,70156859	1,33E-14
BOC	Boc homolog (mouse)	0,700355519	1,55E-14
THBS1	thrombospondin 1	0,700010882	1,62E-14
BGN	biglycan	0,698730548	1,91E-14
FLNA	filamin A, alpha	0,696038289	2,62E-14
MFGE8	milk fat globule-EGF factor 8 protein	0,692796603	3,86E-14
FBLN2	fibulin 2	0,692248194	4,13E-14
LAMA4	laminin, alpha 4	0,692200962	4,15E-14
ENTPD1	ectonucleoside triphosphate diphosphohydrolase 1	0,692101834	4,22E-14
COL8A2	collagen, type VIII, alpha 2	0,691383174	4,57E-14
PDZRN3	PDZ domain containing ring finger 3	0,688952627	6,08E-14
FSTL1	follistatin-like 1	0,688616576	6,33E-14
TPM1	tropomyosin 1 (alpha)	0,688483728	6,44E-14
CNN1	calponin 1, basic, smooth muscle	0,688039785	6,77E-14
MXRA7	matrix-remodelling associated 7	0,687535218	7,19E-14
IGF1	insulin-like growth factor 1 (somatomedin C)	0,687223247	7,46E-14

CPXM1	carboxypeptidase X (M14 family), member 1	0,686910551	7,73E-14
SULF1	sulfatase 1	0,686227943	8,35E-14
FOXF1	forkhead box F1	0,68513021	9,48E-14
MFAP5	microfibrillar associated protein 5	0,682863446	1,23E-13
DAAM2	dishevelled associated activator of morphogenesis 2	0,682596188	1,27E-13
SELM	selenoprotein M	0,682008002	1,35E-13
PLN	phospholamban	0,681901054	1,37E-13
GEM	GTP binding protein overexpressed in skeletal muscle	0,681379933	1,46E-13
RCAN2	regulator of calcineurin 2	0,679209618	1,86E-13
PRKCDBP	protein kinase C, delta binding protein	0,67864204	1,98E-13
RASL12	RAS-like, family 12	0,677872537	2,16E-13
LOC100128178	similar to hCG2041313	0,67700306	2,38E-13
MCAM	melanoma cell adhesion molecule	0,676684866	2,46E-13
A2M	alpha-2-macroglobulin	0,676603169	2,49E-13
DACT1	dapper, antagonist of beta-catenin, homolog 1 (Xenopus laevis)	0,676312533	2,57E-13
RGS2	regulator of G-protein signaling 2, 24kDa	0,675839153	2,71E-13
GAS6	growth arrest-specific 6	0,675206933	2,90E-13
CTHRC1	collagen triple helix repeat containing 1	0,674573318	3,12E-13
GLT8D4	glycosyltransferase 8 domain containing 4	0,673977632	3,33E-13
PODN	podocan	0,673270824	3,60E-13
CPXM2	carboxypeptidase X (M14 family), member 2	0,673163933	3,64E-13
DCHS1	dachsous 1 (Drosophila)	0,672972671	3,72E-13
KCNJ8	potassium inwardly-rectifying channel, subfamily J, member 8	0,671937969	4,16E-13
IFFO1	intermediate filament family orphan 1	0,671098313	4,56E-13
EMCN	endomucin	0,670270164	4,99E-13
SH3RF3	SH3 domain containing ring finger 3	0,669737488	5,28E-13
TSPAN4	tetraspanin 4	0,66730051	6,87E-13
P4HA3	prolyl 4-hydroxylase, alpha polypeptide III	0,666536518	7,46E-13
BNC2	basonuclin 2	0,666353747	7,61E-13
LRRN4CL	LRRN4 C-terminal like	0,665987123	7,91E-13
HEG1	HEG homolog 1 (zebrafish)	0,664571509	9,20E-13
SGK269	NKF3 kinase family member	0,664309429	9,46E-13
CPA3	carboxypeptidase A3 (mast cell)	0,662039289	1,20E-12
COL4A2	collagen, type IV, alpha 2	0,661555618	1,26E-12
FGF7	fibroblast growth factor 7 (keratinocyte growth factor)	0,660510665	1,41E-12
TNS1	tensin 1	0,660369193	1,43E-12
TMEM119	transmembrane protein 119	0,660008752	1,49E-12
FHL1	four and a half LIM domains 1	0,658999391	1,65E-12
FBXL7	F-box and leucine-rich repeat protein 7	0,658197754	1,80E-12
COPZ2	coatomer protein complex, subunit zeta 2	0,656936825	2,05E-12
GAS1	growth arrest-specific 1	0,656747355	2,09E-12
JAM2	junctional adhesion molecule 2	0,656074017	2,24E-12
EFEMP1	EGF-containing fibulin-like extracellular matrix protein 1	0,6551292	2,46E-12
MGP	matrix Gla protein	0,653833302	2,81E-12
GYPC	glycophorin C (Gerbich blood group)	0,653752722	2,83E-12
RECK	reversion-inducing-cysteine-rich protein with kazal motifs	0,653740723	2,84E-12
FERMT2	fermitin family homolog 2 (Drosophila)	0,653077894	3,04E-12
GAS7	growth arrest-specific 7	0,652926089	3,08E-12
CDH5	cadherin 5, type 2 (vascular endothelium)	0,652670712	3,16E-12
NID1	nidogen 1	0,652405919	3,25E-12
BAG2	BCL2-associated athanogene 2	0,652382276	3,26E-12
PECAM1	platelet/endothelial cell adhesion molecule	0,651951531	3,40E-12
GATA6	GATA binding protein 6	0,651734702	3,48E-12
KANK2	KN motif and ankyrin repeat domains 2	0,650343424	4,00E-12
MYH11	myosin, heavy chain 11, smooth muscle	0,649789364	4,23E-12
STARD13	StAR-related lipid transfer (START) domain containing 13	0,647174548	5,50E-12
PDPN	podoplanin	0,646457316	5,90E-12
SRPX	sushi-repeat-containing protein, X-linked	0,645426411	6,54E-12
CD93	CD93 molecule	0,644879544	6,90E-12
PTGDS	prostaglandin D2 synthase 21kDa (brain)	0,643652163	7,79E-12
KCNMB1	potassium large conductance calcium-activated channel, subfamily M, beta member 1	0,643599474	7,83E-12
COL18A1	collagen, type XVIII, alpha 1	0,640821734	1,03E-11
PAPSS2	3'-phosphoadenosine 5'-phosphosulfate synthase 2	0,638715847	1,26E-11
TMEM176B	transmembrane protein 176B	0,638148516	1,33E-11

ASPN	asporin	0,636827301	1,51E-11
GJA4	gap junction protein, alpha 4, 37kDa	0,636764884	1,52E-11
NNMT	nicotinamide N-methyltransferase	0,63675452	1,52E-11
PDGFC	platelet derived growth factor C	0,636044575	1,63E-11
SNORD114-3	small nucleolar RNA, C/D box 114-3	0,634380718	1,91E-11
CSGALNACT1	chondroitin sulfate N-acetylgalactosaminyltransferase 1	0,634004002	1,97E-11
ATP8B2	ATPase, class I, type 8B, member 2	0,63369315	2,03E-11
EMP3	epithelial membrane protein 3	0,633329149	2,11E-11
AQP1	aquaporin 1 (Colton blood group)	0,631486337	2,50E-11
S1PR1	sphingosine-1-phosphate receptor 1	0,630538054	2,74E-11
MOXD1	monooxygenase, DBH-like 1	0,629579843	2,99E-11
FN1	fibronectin 1	0,628417849	3,34E-11
EPB41L2	erythrocyte membrane protein band 4.1-like 2	0,628094494	3,44E-11
MARVELD1	MARVEL domain containing 1	0,628055304	3,45E-11
KCNE4	potassium voltage-gated channel, Isk-related family, member 4	0,626763906	3,89E-11
COLEC12	collectin sub-family member 12	0,624951197	4,59E-11
PARVA	parvin, alpha	0,623574682	5,21E-11
CD200	CD200 molecule	0,622902205	5,54E-11
ZEB1	zinc finger E-box binding homeobox 1	0,622364437	5,82E-11
TMEM158	transmembrane protein 158	0,622022234	6,00E-11
ARHGAP24	Rho GTPase activating protein 24	0,621117122	6,52E-11
HSD17B6	hydroxysteroid (17-beta) dehydrogenase 6 homolog (mouse)	0,62106144	6,55E-11
FNDC1	fibronectin type III domain containing 1	0,620108345	7,14E-11
ACTC1	actin, alpha, cardiac muscle 1	0,617124243	9,33E-11
CTGF	connective tissue growth factor	0,616841262	9,57E-11
LGALS1	lectin, galactoside-binding, soluble, 1	0,616026272	1,03E-10
CCL11	chemokine (C-C motif) ligand 11	0,615906063	1,04E-10
ADCY4	adenylate cyclase 4	0,615253135	1,10E-10
ALPK2	alpha-kinase 2	0,614932039	1,13E-10
RBMS3	RNA binding motif, single stranded interacting protein	0,612223113	1,44E-10
ANTXR2	anthrax toxin receptor 2	0,611191621	1,58E-10
CSRP1	cysteine and glycine-rich protein 1	0,610952504	1,61E-10
LTBP1	latent transforming growth factor beta binding protein 1	0,610915329	1,61E-10
TSHZ3	teashirt zinc finger homeobox 3	0,610613798	1,66E-10
PCDH18	protocadherin 18	0,609952757	1,76E-10
LOXL1	lysyl oxidase-like 1	0,609750415	1,79E-10
RAB23	RAB23, member RAS oncogene family	0,608387583	2,01E-10
PLXDC1	plexin domain containing 1	0,607981941	2,08E-10
ACVRL1	activin A receptor type II-like 1	0,60766816	2,14E-10
TNC	tenascin C	0,607554165	2,16E-10
SYNPO2	synaptopodin 2	0,606145289	2,44E-10
AXL	AXL receptor tyrosine kinase	0,605677223	2,54E-10
DPT	dermatopontin	0,605544162	2,57E-10
COL4A1	collagen, type IV, alpha 1	0,604378204	2,84E-10
PMP22	peripheral myelin protein 22	0,60437081	2,84E-10
DPYSL3	dihydropyrimidinase-like 3	0,603819229	2,98E-10
PPP1R14A	protein phosphatase 1, regulatory (inhibitor) subunit 14A	0,603205273	3,14E-10
PLSCR4	phospholipid scramblase 4	0,602620326	3,30E-10
TIMP1	TIMP metallopeptidase inhibitor 1	0,602442121	3,35E-10
TMEM200A	transmembrane protein 200A	0,601791639	3,54E-10
MITF	microphthalmia-associated transcription factor	0,601536895	3,61E-10
SYNM	synemin, intermediate filament protein	0,601133308	3,74E-10
SGCD	sarcoglycan, delta (35kDa dystrophin-associated glycoprotein)	0,600918302	3,81E-10
ARMCX1	armadillo repeat containing, X-linked 1	0,597835047	4,93E-10
GLIPR1	GLI pathogenesis-related 1	0,596079795	5,70E-10
SORBS1	sorbin and SH3 domain containing 1	0,59595683	5,76E-10
HSPB6	heat shock protein, alpha-crystallin-related, B6	0,595711668	5,88E-10
CYR61	cysteine-rich, angiogenic inducer, 61	0,59541848	6,03E-10
CCDC3	coiled-coil domain containing 3	0,595046363	6,21E-10
ITGA5	integrin, alpha 5 (fibronectin receptor, alpha polypeptide)	0,593667494	6,96E-10
PHLDB1	pleckstrin homology-like domain, family B, member 1	0,593635897	6,98E-10
GLIS2	GLIS family zinc finger 2	0,593624208	6,99E-10
PKIG	protein kinase (cAMP-dependent, catalytic) inhibitor gamma	0,593623349	6,99E-10
DPYSL2	dihydropyrimidinase-like 2	0,591797762	8,12E-10

TIE1	tyrosine kinase with immunoglobulin-like and EGF-like domains 1	0,590839187	8,78E-10
CCND2	cyclin D2	0,590702725	8,87E-10
CH25H	cholesterol 25-hydroxylase	0,589997575	9,40E-10
ZEB2	zinc finger E-box binding homeobox 2	0,589321881	9,93E-10
PPP3CC	protein phosphatase 3 (formerly 2B), catalytic subunit, gamma isoform	0,587828604	1,12E-09
CILP	cartilage intermediate layer protein, nucleotide pyrophosphohydrolase	0,587358731	1,16E-09
PGCP	plasma glutamate carboxypeptidase	0,587144117	1,18E-09
SFRP4	secreted frizzled-related protein 4	0,586227106	1,27E-09
SMTN	smoothelin	0,586203088	1,28E-09
ELN	elastin	0,584902339	1,42E-09
DES	desmin	0,584049326	1,52E-09
TGFBR2	transforming growth factor, beta receptor II (70/80kDa)	0,583567334	1,58E-09
TIMP3	TIMP metallopeptidase inhibitor 3	0,583312725	1,61E-09
LMOD1	leiomodin 1 (smooth muscle)	0,582714603	1,69E-09
ITGA11	integrin, alpha 11	0,582153519	1,76E-09
CLEC11A	C-type lectin domain family 11, member A	0,581933546	1,79E-09
PTGER3	prostaglandin E receptor 3 (subtype EP3)	0,580987195	1,93E-09
ADH1B	alcohol dehydrogenase 1B (class I), beta polypeptide	0,57833498	2,38E-09
IFITM2	interferon induced transmembrane protein 2 (1-8D)	0,577067005	2,63E-09
WFDC1	WAP four-disulfide core domain 1	0,576751926	2,69E-09
OGN	osteoglycin	0,576367488	2,77E-09
TWIST2	twist homolog 2 (Drosophila)	0,576272027	2,79E-09
TMEM176A	transmembrane protein 176A	0,576061504	2,84E-09
JAM3	junctional adhesion molecule 3	0,575918584	2,87E-09
ROBO4	roundabout homolog 4, magic roundabout (Drosophila)	0,574938086	3,10E-09
HTRA3	HtrA serine peptidase 3	0,574801112	3,13E-09
SDC2	syndecan 2	0,574559575	3,19E-09
FAP	fibroblast activation protein, alpha	0,574482426	3,21E-09
FGFR1	fibroblast growth factor receptor 1	0,573408196	3,49E-09
GALNTL2	UDP-N-acetyl-alpha-D-galactosamine:polypeptide N-acetylgalactosaminyltransferase-like 2	0,573226005	3,53E-09
RGS5	regulator of G-protein signaling 5	0,572984174	3,60E-09
CHRDL2	chordin-like 2	0,572927581	3,62E-09
GGTA1	glycoprotein, alpha-galactosyltransferase 1	0,572918991	3,62E-09
ZAK	sterile alpha motif and leucine zipper containing kinase AZK	0,571340599	4,08E-09
HDC	histidine decarboxylase	0,571297795	4,10E-09
ZNF469	zinc finger protein 469	0,570673418	4,30E-09
DARC	Duffy blood group, chemokine receptor	0,570127381	4,48E-09
VCL	vinculin	0,570006025	4,52E-09
TPSAB1	tryptase alpha/beta 1	0,568903664	4,91E-09
GNG11	guanine nucleotide binding protein (G protein), gamma 11	0,568383389	5,11E-09
GPR116	G protein-coupled receptor 116	0,567912513	5,30E-09
CASQ2	calsequestrin 2 (cardiac muscle)	0,567868094	5,31E-09
APLNR	apelin receptor	0,566980195	5,68E-09
STOM	stomatin	0,566708987	5,80E-09
NDN	necdin homolog (mouse)	0,566324385	5,97E-09
TPST1	tyrosylprotein sulfotransferase 1	0,566100178	6,07E-09
PLAC9	placenta-specific 9	0,565741904	6,23E-09
SLIT2	slit homolog 2 (Drosophila)	0,565639369	6,28E-09
KLF2	Kruppel-like factor 2 (lung)	0,565582184	6,31E-09
ACOX2	acyl-Coenzyme A oxidase 2, branched chain	0,565570091	6,31E-09
TCF21	transcription factor 21	0,56496174	6,61E-09
SHANK3	SH3 and multiple ankyrin repeat domains 3	0,563567122	7,33E-09
VASN	vasorin	0,563550885	7,34E-09
EGR2	early growth response 2	0,563476697	7,38E-09
CRYAB	crystallin, alpha B	0,563025045	7,63E-09
HSPA12A	heat shock 70kDa protein 12A	0,560831946	8,98E-09
SDPR	serum deprivation response (phosphatidylserine binding protein)	0,56050601	9,19E-09
NEXN	nexilin (F actin binding protein)	0,559811665	9,68E-09
FKBP7	FK506 binding protein 7	0,559586469	9,84E-09

SERPINE2	serpin peptidase inhibitor, clade E (nexin, plasminogen activator inhibitor type 1), member 2	0,55938172	9,99E-09
TUBB6	tubulin, beta 6	0,558410911	1,07E-08
SGK493	protein kinase-like protein SgK493	0,558280381	1,08E-08
ASAM	adipocyte-specific adhesion molecule	0,558180827	1,09E-08
NEGR1	neuronal growth regulator 1	0,557880974	1,11E-08
SYNC	syncoilin, intermediate filament protein	0,557859745	1,12E-08
CMAH	cytidine monophosphate-N-acetylneuraminic acid hydroxylase (CMP-N-acetylneuraminate monooxygenase) pseudogene	0,557740048	1,13E-08
SOX17	SRY (sex determining region Y)-box 17	0,557646571	1,13E-08
EHD2	EH-domain containing 2	0,557406983	1,15E-08
NTM	neurotrimin	0,557278922	1,16E-08
PID1	phosphotyrosine interaction domain containing 1	0,557075964	1,18E-08
C13orf33	chromosome 13 open reading frame 33	0,556861029	1,20E-08
CALCRL	calcitonin receptor-like	0,555652083	1,31E-08
SNED1	sushi, nidogen and EGF-like domains 1	0,555268318	1,35E-08
TMEM204	transmembrane protein 204	0,554336779	1,44E-08
F13A1	coagulation factor XIII, A1 polypeptide	0,553570218	1,52E-08
ITIH5	inter-alpha (globulin) inhibitor H5	0,553084172	1,58E-08
BHMT2	betaine-homocysteine methyltransferase 2	0,551197147	1,81E-08
CNPY4	canopy 4 homolog (zebrafish)	0,550326117	1,92E-08
LAMC1	laminin, gamma 1 (formerly LAMB2)	0,55004351	1,96E-08
SH3BGRL	SH3 domain binding glutamic acid-rich protein like	0,549836079	1,99E-08
EDNRB	endothelin receptor type B	0,548650257	2,16E-08
KDR	kinase insert domain receptor (a type III receptor tyrosine kinase)	0,548460468	2,19E-08
C7orf58	chromosome 7 open reading frame 58	0,548436084	2,20E-08
PRELP	proline/arginine-rich end leucine-rich repeat protein	0,547465244	2,35E-08
GUCY1B3	guanylate cyclase 1, soluble, beta 3	0,546915526	2,45E-08
RAMP3	receptor (G protein-coupled) activity modifying protein 3	0,546675249	2,49E-08
PLXND1	plexin D1	0,546644649	2,49E-08
ACTN1	actinin, alpha 1	0,546318174	2,55E-08
EFHA2	EF-hand domain family, member A2	0,546297016	2,55E-08
DOK5	docking protein 5	0,546173164	2,58E-08
RCN3	reticulocalbin 3, EF-hand calcium binding domain	0,546117536	2,59E-08
SHE	Src homology 2 domain containing E	0,545798957	2,64E-08
BIN1	bridging integrator 1	0,545758579	2,65E-08
ITGBL1	integrin, beta-like 1 (with EGF-like repeat domains)	0,545750329	2,65E-08
RFTN2	raftlin family member 2	0,545435971	2,71E-08
WIPF1	WAS/WASL interacting protein family, member 1	0,545210444	2,76E-08
NXN	nucleoredoxin	0,544878073	2,82E-08
GPR183	G protein-coupled receptor 183	0,544836551	2,83E-08
ADAMTS9	ADAM metallopeptidase with thrombospondin type 1 motif, 9	0,544648522	2,87E-08
ADRA2A	adrenergic, alpha-2A-, receptor	0,54451721	2,89E-08
FIBIN	fin bud initiation factor homolog (zebrafish)	0,544129905	2,97E-08
ADAM33	ADAM metallopeptidase domain 33	0,543432576	3,12E-08
FXYD1	FXYD domain containing ion transport regulator 1	0,543335649	3,14E-08
DKK3	dickkopf homolog 3 (Xenopus laevis)	0,542489243	3,33E-08
HSPG2	heparan sulfate proteoglycan 2	0,542199227	3,40E-08
ADAMTS1	ADAM metallopeptidase with thrombospondin type 1 motif, 1	0,541801223	3,49E-08
STX2	syntaxin 2	0,54177494	3,50E-08
RSPO3	R-spondin 3 homolog (Xenopus laevis)	0,541388465	3,60E-08
MSN	moesin	0,541098464	3,67E-08
FLNC	filamin C, gamma	0,540936344	3,71E-08
FAM26E	family with sequence similarity 26, member E	0,540926405	3,71E-08
CD34	CD34 molecule	0,540792703	3,75E-08
PDLIM2	PDZ and LIM domain 2 (mystique)	0,540433667	3,84E-08
AOX1	aldehyde oxidase 1	0,540261487	3,89E-08
MEF2C	myocyte enhancer factor 2C	0,540024291	3,95E-08
ARHGEF17	Rho guanine nucleotide exchange factor (GEF) 17	0,539705468	4,04E-08
SELP	selectin P (granule membrane protein 140kDa, antigen CD62)	0,539515879	4,09E-08
LRRC17	leucine rich repeat containing 17	0,539327915	4,14E-08
DLC1	deleted in liver cancer 1	0,539289894	4,15E-08
HPGDS	hematopoietic prostaglandin D synthase	0,538420321	4,41E-08
LYVE1	lymphatic vessel endothelial hyaluronan receptor 1	0,53768625	4,64E-08

TENC1	tensin like C1 domain containing phosphatase (tensin 2)	0,53768339	4,64E-08
PPP1R12B	protein phosphatase 1, regulatory (inhibitor) subunit 12B	0,537588776	4,67E-08
MEOX1	mesenchyme homeobox 1	0,536888781	4,90E-08
ALDH2	aldehyde dehydrogenase 2 family (mitochondrial)	0,536127999	5,16E-08
PTGFR	prostaglandin F receptor (FP)	0,536101886	5,16E-08
CAV1	caveolin 1, caveolae protein, 22kDa	0,534748559	5,66E-08
EPHA3	EPH receptor A3	0,534477104	5,77E-08
GSN	gelsolin (amyloidosis, Finnish type)	0,533163023	6,30E-08
HMCN1	hemicentin 1	0,532545377	6,57E-08
GUCY1A3	guanylate cyclase 1, soluble, alpha 3	0,532180971	6,73E-08
MST150	MSTP150	0,532136101	6,75E-08
ITM2A	integral membrane protein 2A	0,531970097	6,83E-08
CCPG1	cell cycle progression 1	0,531759089	6,92E-08
ILK	integrin-linked kinase	0,531706704	6,95E-08
RASA3	RAS p21 protein activator 3	0,530911475	7,33E-08
C2orf40	chromosome 2 open reading frame 40	0,530643261	7,46E-08
COMP	cartilage oligomeric matrix protein	0,530286972	7,64E-08
IL33	interleukin 33	0,530146407	7,71E-08
hCG_1806964	hypothetical LOC401093	0,530065103	7,75E-08
CYTL1	cytokine-like 1	0,52971609	7,93E-08
MEIS1	Meis homeobox 1	0,529687612	7,95E-08
PTGS1	prostaglandin-endoperoxide synthase 1 (prostaglandin G/H synthase and cyclooxygenase)	0,529685323	7,95E-08
CYYR1	cysteine/tyrosine-rich 1	0,52898429	8,33E-08
CYBRD1	cytochrome b reductase 1	0,52893539	8,36E-08
PLA2G5	phospholipase A2, group V	0,528837626	8,41E-08
C1QTNF1	C1q and tumor necrosis factor related protein 1	0,528689382	8,49E-08
P2RX1	purinergic receptor P2X, ligand-gated ion channel, 1	0,528175519	8,79E-08
C15orf5	chromosome 15 open reading frame 5	0,528140796	8,81E-08
SOBP	sine oculis binding protein homolog (Drosophila)	0,527684849	9,08E-08
PAM	peptidylglycine alpha-amidating monooxygenase	0,527215358	9,37E-08
LAMB2	laminin, beta 2 (laminin S)	0,527124827	9,42E-08
RBPMS2	RNA binding protein with multiple splicing 2	0,526735798	9,67E-08
FLRT2	fibronectin leucine rich transmembrane protein 2	0,526125204	1,01E-07
COL21A1	collagen, type XXI, alpha 1	0,526120876	1,01E-07
PDGFRL	platelet-derived growth factor receptor-like	0,525328773	1,06E-07
HSPA12B	heat shock 70kD protein 12B	0,524083598	1,15E-07
MMRN1	multimerin 1	0,523745448	1,18E-07
SPON2	spondin 2, extracellular matrix protein	0,523533954	1,19E-07
GPC6	glypican 6	0,522093544	1,31E-07
PRDM6	PR domain containing 6	0,522071054	1,31E-07
FXYD6	FXYD domain containing ion transport regulator 6	0,521560915	1,36E-07
COL13A1	collagen, type XIII, alpha 1	0,521159737	1,39E-07
RUNX1T1	runt-related transcription factor 1, translocated to, 1 (cyclin D-related)	0,520846285	1,42E-07
MAP1A	microtubule-associated protein 1A	0,5204148	1,46E-07
FAM38B	family with sequence similarity 38, member B	0,520125452	1,49E-07
MAP1B	microtubule-associated protein 1B	0,519793017	1,52E-07
KLHL5	kelch-like 5 (Drosophila)	0,518928745	1,61E-07
FGL2	fibrinogen-like 2	0,518590456	1,64E-07
CCL14	chemokine (C-C motif) ligand 14	0,518570734	1,64E-07
CLDN5	claudin 5	0,517711127	1,74E-07
HSD11B1	hydroxysteroid (11-beta) dehydrogenase 1	0,517657965	1,74E-07
BDKRB2	bradykinin receptor B2	0,517630922	1,75E-07
ALDH1B1	aldehyde dehydrogenase 1 family, member B1	0,517625629	1,75E-07
KLF9	Kruppel-like factor 9	0,517624245	1,75E-07
HSPB2	heat shock 27kDa protein 2	0,516638884	1,86E-07
SCRG1	stimulator of chondrogenesis 1	0,516413187	1,89E-07
OMD	osteomodulin	0,515893431	1,95E-07
CFL2	cofilin 2 (muscle)	0,515645403	1,98E-07
ITGA7	integrin, alpha 7	0,515169828	2,04E-07
SIDT2	SID1 transmembrane family, member 2	0,514918722	2,08E-07
LRRC70	leucine rich repeat containing 70	0,514726982	2,10E-07
TRAM2	translocation associated membrane protein 2	0,514254775	2,17E-07
C7	complement component 7	0,513881388	2,22E-07

CLIP3	CAP-GLY domain containing linker protein 3	0,513399374	2,29E-07
CSGALNACT2	chondroitin sulfate N-acetylgalactosaminyltransferase 2	0,512996364	2,34E-07
POPDC2	popeye domain containing 2	0,51272841	2,38E-07
COL12A1	collagen, type XII, alpha 1	0,512580046	2,41E-07
CHSY3	chondroitin sulfate synthase 3	0,512407764	2,43E-07
SEMA3G	sema domain, immunoglobulin domain (Ig), short basic domain, secreted, (semaphorin) 3G	0,511994015	2,50E-07
GIMAP8	GTPase, IMAP family member 8	0,511856907	2,52E-07
SLIT3	slit homolog 3 (Drosophila)	0,511627563	2,56E-07
P2RY14	purinergic receptor P2Y, G-protein coupled, 14	0,511490725	2,58E-07
CCDC102B	coiled-coil domain containing 102B	0,51001874	2,83E-07
ADAMTS5	ADAM metallopeptidase with thrombospondin type 1 motif, 5	0,509953961	2,84E-07
CHN1	chimerin (chimaerin) 1	0,509773963	2,87E-07
ESAM	endothelial cell adhesion molecule	0,509302766	2,96E-07
PAMR1	peptidase domain containing associated with muscle regeneration 1	0,508981832	3,01E-07
LIMS2	LIM and senescent cell antigen-like domains 2	0,508558389	3,10E-07
SORBS3	sorbin and SH3 domain containing 3	0,508345452	3,14E-07
GFPT2	glutamine-fructose-6-phosphate transaminase 2	0,508112381	3,18E-07
PLVAP	plasmalemma vesicle associated protein	0,507827654	3,24E-07
UACA	uveal autoantigen with coiled-coil domains and ankyrin repeats	0,507646312	3,28E-07
ADAMTS8	ADAM metallopeptidase with thrombospondin type 1 motif, 8	0,507128295	3,38E-07
FRZB	frizzled-related protein	0,506782521	3,46E-07
SPRY1	sprouty homolog 1, antagonist of FGF signaling (Drosophila)	0,506346355	3,55E-07
ARHGAP1	Rho GTPase activating protein 1	0,505526681	3,73E-07
MAOB	monoamine oxidase B	0,505526349	3,73E-07
ADAM12	ADAM metallopeptidase domain 12	0,504914852	3,88E-07
VWF	von Willebrand factor	0,504220178	4,05E-07
PTGIS	prostaglandin I2 (prostacyclin) synthase	0,504130967	4,07E-07
CHST15	carbohydrate (N-acetylgalactosamine 4-sulfate 6-O) sulfotransferase 15	0,503948825	4,11E-07
MYCT1	myc target 1	0,503574916	4,21E-07
LAYN	layilin	0,503020949	4,35E-07
KCTD10	potassium channel tetramerisation domain containing 10	0,502514749	4,49E-07
LPPR4	plasticity related gene 1	0,501818702	4,69E-07
KIAA1644	KIAA1644	0,501381163	4,81E-07
FAM129A	family with sequence similarity 129, member A	0,501236788	4,85E-07
EGR1	early growth response 1	0,500549969	5,06E-07
PDE1A	phosphodiesterase 1A, calmodulin-dependent	0,500427762	5,10E-07
PAPPA	pregnancy-associated plasma protein A, pappalysin 1	0,500406979	5,11E-07
ABCA9	ATP-binding cassette, sub-family A (ABC1), member 9	0,500191915	5,17E-07
C1orf226	chromosome 1 open reading frame 226	-0,502854036	4,40E-07
ZSWIM3	zinc finger, SWIM-type containing 3	-0,510648578	2,72E-07
DCAF7	DDB1 and CUL4 associated factor 7	-0,518652155	1,64E-07
C21orf49	chromosome 21 open reading frame 49	-0,523834329	1,17E-07
DARS2	aspartyl-tRNA synthetase 2, mitochondrial	-0,5249644	1,09E-07
MARVELD2	MARVEL domain containing 2	-0,525110079	1,08E-07
MCM3APAS	MCM3AP antisense RNA (non-protein coding)	-0,527444169	9,22E-08
DUSP12	dual specificity phosphatase 12	-0,532874915	6,42E-08
OVOL2	ovo-like 2 (Drosophila)	-0,537186858	4,80E-08
ZNF672	zinc finger protein 672	-0,537374006	4,74E-08
MTMR4	myotubularin related protein 4	-0,554562318	1,42E-08
APITD1	apoptosis-inducing, TAF9-like domain 1	-0,558105609	1,10E-08
FKBP4	FK506 binding protein 4, 59kDa	-0,558742831	1,05E-08
C1orf172	chromosome 1 open reading frame 172	-0,569760796	4,60E-09
C1orf96	chromosome 1 open reading frame 96	-0,570734397	4,28E-09

Supplementary Table 2: Functional pathways and gene ontology annotations relative to biological processes significantly enriched in genes positively correlated with *DCN* in human MIBC.

Genes positively correlated with *DCN* listed in supplementary table 1 were analyzed using DAVID functional annotation tool. Corrected p-value used were $p<1.10^{-2}$ and $p<1.10^{-3}$ for functional pathways and for gene ontology annotations, respectively.

Supplementary Table 2

Supplementary Table2: Functionnal pathways and gene ontology annotations relative to biological process significantly (corrected p-value p<1E-2 and p<1.E-3 resepctivily) enriched in genes positively correlated to DCN in human MIBC (supplementary Table1) identified using DAVID functionnal annotation tool.

Input / Pathway genes	Pathway	Corrected p-value	Input Genes
25/80	ECM-receptor interaction	1.85E-10	COL1A1, COL1A2, COL3A1 , COL4A1, COL4A2, COL5A1, COL5A2, COL6A1, COL6A2, COL6A3, COMP, FN1, HSPG2, ITGA11, ITGA5, ITGA7, LAMA2, LAMA4, LAMB2, LAMC1, SDC2, THBS1, THBS2, TNC,VWF
38/194	Focal adhesion	4.31E-10	ACTN1, CAV1, CCND2, COL1A1, COL1A2, COL3A1, COL4A1, COL4A2, COL5A1, COL5A2, COL6A1, COL6A2, COL6A3, COMP, FLNA, FLNC, FN1, IGF1, ILK, ITGA11, ITGA5, ITGA7, KDR, LAMA2, LAMA4, LAMB2, LAMC1, MYL9, MYLK, PARVA, PDGFC, PDGFRA, PDGFRB, THBS1, THBS2,VCL, VWF
14/109	Leukocyte transendothelial migration	4.93E-05	ACTN1, CDH5, CLDN5, CXCL12, ESAM, JAM2, JAM3, MMP2, MSN, MYL9, PECAM1, THY1, VCAM1, VCL
17/204	Regulation of actin cytoskeleton	0.0013918	ACTN1, BDKRB2, CFL2, FGF7, FGFR1, FN1, GSN, ITGA11, ITGA5, ITGA7, MSN, MYL9, MYLK, PDGFC, PDGFRA, PDGFRB, VCL
12/125	Cell adhesion molecules (CAMs)	0.0057623	CD34, CDH5, CLDN5, ESAM, JAM2, JAM3, NEGR1, PECAM1, SDC2, SELP, VCAM1, VCAN

GO id	GO Term	Corrected P-value	Genes
GO:0007155	cell adhesion	1.17E-34	DLC1, AEBP1, COL21A1, IGFBP7, FERMT2, CLDN5, POSTN, MMRN1, CXCL12, DDR2, VCL, SRPX, S1PR1, CD93, CTGF, ILK, COL12A1, ESAM, MSN, SPON2, NEGR1, BOC, CYR61, SPON1, PDPN, MGP, ACTN1, CPXM2, MFGE8, THY1, CD34, CPXM1, VCAN, LAMC1, TGFB1I1, JAM2, ADAM12, MFAP4, AOC3, PARVA, EMCN, TNC, COL3A1, ITGA11, DCHS1, CDH5, ITGBL1, VCAM1, ISLR, SORBS3, LAMB2, SORBS1, COMP, COL6A3, COL6A2, COL6A1, ENTPD1, THBS1, THBS2, COL8A2, FN1, DPT, COL18A1, FLRT2, SELP, COL13A1, COL15A1, HSPG2, NID1, NID2, ECM2, MCAM, COL16A1, COL5A1, PCDH18, EMILIN1, CCL11, LAMA2, VWF, LYVE1, LAMA4, OMD, COL14A1, SNED1, ITGA5, FBLN5, ITGA7, PECAM1, ANTXR1, NTM, CDH11
GO:0022610	biological adhesion	1.32E-34	DLC1, AEBP1, COL21A1, IGFBP7, FERMT2, CLDN5, POSTN, MMRN1, CXCL12, DDR2, VCL, SRPX, S1PR1, CD93, CTGF, ILK, COL12A1, ESAM, MSN, SPON2, NEGR1, BOC, CYR61, SPON1, PDPN, MGP, ACTN1, CPXM2, MFGE8, THY1, CD34, CPXM1, VCAN, LAMC1, TGFB1I1, JAM2, ADAM12, MFAP4, AOC3, PARVA, EMCN, TNC, COL3A1, ITGA11, DCHS1, CDH5, ITGBL1, VCAM1, ISLR, SORBS3, LAMB2, SORBS1, COMP, COL6A3, COL6A2, COL6A1, ENTPD1, THBS1, THBS2, COL8A2, FN1, DPT, COL18A1, FLRT2, SELP, COL13A1, COL15A1, HSPG2, NID1, NID2, ECM2, MCAM, COL16A1, COL5A1, PCDH18, EMILIN1, CCL11, LAMA2, VWF, LYVE1, LAMA4, OMD, COL14A1, SNED1, ITGA5, FBLN5, ITGA7, PECAM1, ANTXR1, NTM, CDH11
GO:0030198	extracellular matrix organization	5.72E-20	LUM, ELN, COL3A1, POSTN, DCN, CRISPLD2, FOXF1, ILK, COL6A2, COL12A1, COL8A2, DPT, CYR61, COL18A1, RECK, COL4A2, CCDC80, HSPG2, NID1, ECM2, COL5A2, COL5A1, EMILIN1, CSGALNACT1, COL14A1, FBLN5, COL1A2, PDGFRA, MYH11, COL1A1, LAMC1
GO:0043062	extracellular structure organization	6.41E-16	TNC, LUM, COL3A1, ELN, POSTN, DCN, CRISPLD2, FOXF1, ILK, COL6A2, COL12A1, COL8A2, DPT, CYR61, RECK, COL18A1, COL4A2, MAP1B, CCDC80, HSPG2, NID1, ECM2, COL5A2, COL5A1, EMILIN1, CSGALNACT1, COL14A1, FBLN5, COL1A2, PDGFRA, MYH11, COL1A1, LAMC1
GO:0001944	vasculature development	2.19E-13	EMCN, CAV1, ACVRL1, COL3A1, PRRX1, GJA4, MMP2, CXCL12, CDH5, EDNRA, TCF21, S1PR1, CTGF, FOXF1, ROBO4, SOX17, PLXND1, THBS1, CYR61, RECK, COL18A1, SELP, PDPN, TGFBR2, COL15A1, ARHGAP24, COL5A1, SLIT2, THY1, KDR, LAMA4, BGN, PLXDC1, ITGA7, COL1A2, ZFPM2, COL1A1
GO:0001568	blood vessel development	3.50E-12	EMCN, CAV1, ACVRL1, COL3A1, PRRX1, GJA4, MMP2, CXCL12, CDH5, EDNRA, S1PR1, CTGF, FOXF1, ROBO4, SOX17, PLXND1, THBS1, CYR61, RECK, COL18A1, SELP, TGFBR2, COL15A1, ARHGAP24, COL5A1, SLIT2, THY1, KDR, LAMA4, BGN, PLXDC1, ITGA7, COL1A2, ZFPM2, COL1A1
GO:0031589	cell-substrate adhesion	1.73E-10	DLC1, COL13A1, COL3A1, ITGA11, ACTN1, NID1, NID2, ECM2, ITGBL1, THY1, VWF, LYVE1, SORBS3, SNED1, SORBS1, CTGF, FBLN5, ILK, ITGA7, ANTXR1, LAMC1, FN1
GO:0009611	response to wounding	1.09E-07	TPST1, C7, A2M, FGF7, ACVRL1, F13A1, TNC, COL3A1, TGFB3, C1R, C1S, BDKRB2, MMRN1, TIMP3, TPM1, HMCN1, LAMB2, CTGF, GSN, THBS1, ENTPD1, PAPSS2, FN1, SELP, PTGER3, PDPN, EFEMP2, MAP1B, TGFBR2, IGF1, COL5A1, EPHA3, CCL11, VWF, LYVE1, P2RX1, PLSCR4, DARC, ITGA5, FBLN5, AOX1, PDGFRA, VCAN, AOC3
GO:0007160	cell-matrix adhesion	2.89E-07	DLC1, COL13A1, COL3A1, ITGA11, ACTN1, NID1, NID2, ECM2, ITGBL1, THY1, LYVE1, SNED1, SORBS1, CTGF, FBLN5, ILK, ITGA7, FN1
GO:0035295	tube development	3.30E-07	DLC1, FGF7, TGFB3, ZEB2, GREM1, CXCL12, EDNRA, TCF21, SPRY1, OVOL2, CRISPLD2, GATA6, CTGF, FOXF1, ILK, PLXND1, CYR61, PDPN, TGFBR2, IGF1, MGP, SHANK3, SLIT2, KDR, HSD11B1, PDGFRA, ZFPM2
GO:0048514	blood vessel morphogenesis	7.11E-07	CAV1, EMCN, ACVRL1, PRRX1, CXCL12, EDNRA, S1PR1, CTGF, FOXF1, ROBO4, SOX17, PLXND1, THBS1, CYR61, COL18A1, SELP, TGFBR2, COL15A1, ARHGAP24, SLIT2, THY1, KDR, BGN, PLXDC1, ITGA7, ZFPM2
GO:0030334	regulation of cell migration	1.38E-06	DLC1, COL18A1, SELP, ACVRL1, PDPN, IGF1, GREM1, NEXN, CXCL12, TPM1, THY1, VCL, KDR, LAMA2, LAMA4, S1PR1, SERPINE2, ILK, PDGFRA, ROBO4, PDGFRB, TIE1, THBS1
GO:0040012	regulation of locomotion	2.95E-06	DLC1, COL18A1, SELP, ACVRL1, PDPN, IGF1, GREM1, NEXN, CXCL12, TPM1, SLIT2, THY1, VCL, KDR, LAMA2, LAMA4, S1PR1, SERPINE2, ILK, PDGFRA, ROBO4, PDGFRB, TIE1, THBS1
GO:0051270	regulation of cell motion	3.27E-06	DLC1, COL18A1, SELP, ACVRL1, PDPN, ACTN1, IGF1, GREM1, NEXN, CXCL12, TPM1, THY1, VCL, KDR, LAMA2, LAMA4, S1PR1, SERPINE2, ILK, PDGFRA, ROBO4, PDGFRB, TIE1, THBS1
GO:0042060	wound healing	1.41E-05	FGF7, ACVRL1, F13A1, EFEMP2, TGFBR2, COL3A1, TGFB3, IGF1, MMRN1, TIMP3, TPM1, COL5A1, VWF, HMCN1, PLSCR4, P2RX1, ITGA5, GSN, FBLN5, PDGFRA, ENTPD1, PAPSS2, FN1
GO:0007517	muscle organ development	8.81E-05	MEF2C, CAV1, AEBP1, ACTC1, CRYAB, FHL1, TNC, ELN, ITGA11, HSPG2, IGF1, TPM1, LAMA2, SMTN, TAGLN, GATA6, PLN, ITGA7, COL6A3, MYH11, SGCD, PDGFRB, ZFPM2
GO:0001525	angiogenesis	1.46E-04	FGFR1, CALCRL, THY1, KDR, TIE1, CTGF, ACVRL1, ITGA5, SOX17, MEIS1, COL8A2, HSPG2, CD34, PLXDC1, COL4A2, COL4A1, MMP2, ROBO4, COL18A1, ARHGAP24, MCAM, PLXND1, PARVA, JAM3, S1PR1, GPR124,FN1, MFGE8, THBS1, CYR61,COL15A1
GO:0042127	regulation of cell proliferation	2.75E-04	DLC1, FGFR1, CAV1, FGF7, ACVRL1, NDN, IGFBP7, MITF, PTGS1, PRRX1, TGFB3, BDKRB2, ZEB1, TIMP2, TENC1, DDR2, CDH5, TIMP1, VCAM1, EDNRA, EDNRB, S1PR1, OVOL2, ILK, ADRA2A, PDGFC, CALCRL, THBS1, DPT, COL18A1, TGFBR2, IGF1, MFGE8, GAS1, SPARC, CLEC11A, KDR, ADAMTS8, CCL14, CCND2, PDGFRA, PDGFRB, WFDC1, ADAMTS1, TGFB1I1, LAMC1, EMP3, PMP22
GO:0030036	actin cytoskeleton organization	3.00E-04	RHOJ, DLC1, ACTC1, CALD1, ELN, FERMT2, PDLIM3, ARHGEF17, ACTN1, DAAM2, TPM1, GAS7, FLNA, EPB41L2, SORBS1, GSN, ADRA2A, MYH11, ANTXR1, CNN1, WIPF1, EHD2, PARVA
GO:0035239	tube morphogenesis	4.79E-04	DLC1, PDPN, TGFBR2, IGF1, MGP, ZEB2, GREM1, CXCL12, SHANK3, KDR, EDNRA, TCF21, OVOL2, FOXF1, ILK, PLXND1, CYR61
GO:0006928	cell motion	8.61E-04	CTHRC1, ACVRL1, NDN, CALD1, VIM, ITGA11, ZEB2, CXCL12, TPM1, VCL, VCAM1, EDNRB, OVOL2, CTGF, ADRA2A, MSN, SPON2, THBS1, FN1, SELP, EGR2, IGF1, GAS1, COL5A1, SLIT2, KDR, SLIT3, LYVE1, TNS1, CD34, ITGA5, PDGFRB, VCAN, LAMC1
GO:0030029	actin filament-based process	9.18E-04	RHOJ, DLC1, ACTC1, CALD1, ELN, FERMT2, PDLIM3, ARHGEF17, ACTN1, DAAM2, TPM1, GAS7, FLNA, EPB41L2, SORBS1, GSN, ADRA2A, MYH11, ANTXR1, CNN1, WIPF1, EHD2, PARVA
GO:0001763	morphogenesis of a branching structure	9.98E-04	COL13A1, TGFBR2, IGF1, MGP, GREM1, CXCL12, KDR, EDNRA, TCF21, FOXF1, ILK, PLXND1, CYR61
GO:0030199	collagen fibril organization	0.001	COL14A1, LUM, COL3A1, COL1A2, COL12A1, COL1A1, COL5A2, COL5A1, DPT

Supplementary Table 3: Affymetrix U133plus2.0 microarray data in 162 human bladder tumors and 4 normal samples, for genes correlated with *DCN* in MIBC (pearson R>0.5).

Affymetrix U133plus2.0 microarray data were normalized using the RMA method together with BrainArray custom chip description files (CDF) for genes correlated with *DCN* in MIBC (pearson R>0.5). Data obtained in 162 bladder tumors and 4 normal samples.

see accompanying excel file.
